# CDP-Diacylglycerol Synthases (CDS): Gateway to Phosphatidylinositol and Cardiolipin Synthesis

**DOI:** 10.3389/fcell.2020.00063

**Published:** 2020-02-07

**Authors:** Nicholas J. Blunsom, Shamshad Cockcroft

**Affiliations:** Division of Biosciences, Department of Neuroscience, Physiology and Pharmacology, University College London, London, United Kingdom

**Keywords:** phospholipase C, lipid synthesis, mitochondria, CDP-diacylglycerol, phosphatidic acid, endoplasmic reticulum, TAMM41, phosphatidylinositol

## Abstract

Cytidine diphosphate diacylglycerol (CDP-DAG) is a key intermediate in the synthesis of phosphatidylinositol (PI) and cardiolipin (CL). Both PI and CL have highly specialized roles in cells. PI can be phosphorylated and these phosphorylated derivatives play major roles in signal transduction, membrane traffic, and maintenance of the actin cytoskeletal network. CL is the signature lipid of mitochondria and has a plethora of functions including maintenance of cristae morphology, mitochondrial fission, and fusion and for electron transport chain super complex formation. Both lipids are synthesized in different organelles although they share the common intermediate, CDP-DAG. CDP-DAG is synthesized from phosphatidic acid (PA) and CTP by enzymes that display CDP-DAG synthase activities. Two families of enzymes, CDS and TAMM41, which bear no sequence or structural relationship, have now been identified. TAMM41 is a peripheral membrane protein localized in the inner mitochondrial membrane required for CL synthesis. CDS enzymes are ancient integral membrane proteins found in all three domains of life. In mammals, they provide CDP-DAG for PI synthesis and for phosphatidylglycerol (PG) and CL synthesis in prokaryotes. CDS enzymes are critical for maintaining phosphoinositide levels during phospholipase C (PLC) signaling. Hydrolysis of PI (4,5) bisphosphate by PLC requires the resynthesis of PI and CDS enzymes catalyze the rate-limiting step in the process. In mammals, the protein products of two CDS genes (CDS1 and CDS2) localize to the ER and it is suggested that CDS2 is the major CDS for this process. Expression of CDS enzymes are regulated by transcription factors and CDS enzymes may also contribute to CL synthesis in mitochondria. Studies of CDS enzymes in protozoa reveal spatial segregation of CDS enzymes from the rest of the machinery required for both PI and CL synthesis identifying a key gap in our understanding of how CDP-DAG can cross the different membrane compartments in protozoa and in mammals.

## Introduction

**FIGURE 1:**
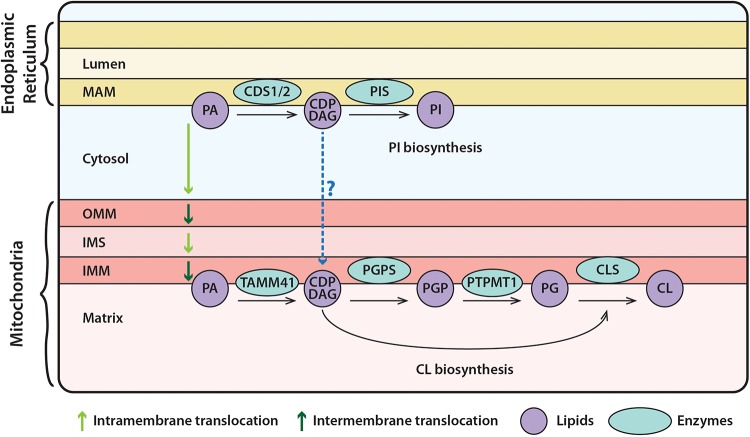
Synthesis of phosphatidylinositol (PI) at the ER and of cardiolipin (CL) in mitochondria. PA, in the ER, is converted to CDP-DAG via the CDS enzymes. CDP-DAG is used by PIS, to synthesize PI. PA can be transferred from the ER to mitochondria, where it is transported across the OMM and IMS to the IMM. Here, TAMM41 utilizes PA to make CDP-DAG, then PGP via PGPS, and PG via PTPMT1. Finally, CL is made by CLS using PG and another molecule of CDP-DAG. It could be the case the CDP-DAG is transported across the cytosol from the ER to mitochondria, where it can be utilized for CL synthesis as discussed in the text. PA, phosphatidic acid; PIS, PI synthase; TAMM41, enzyme with CDS activity; PGPS, phosphatidylglycerolphosphate synthase; CLS, cardiolipin synthase; PI, phosphatidylinositol; CDP-DAG, CDP-diacylglycerol; PGP, phosphatidylglycerol phosphate; PTPMT1, protein tyrosine phosphatase mitochondrial 1; PG, phosphatidylglycerol; CL, cardiolipin; MAM, mitochondrial associated membranes; OMM, outer mitochondrial membrane; IMS, inner mitochondrial space; IMM, inner mitochondrial membrane.

Phosphatidylinositol (and its phosphorylated derivatives) and CL are two anionic phospholipids that perform a plethora of essential functions in cells. Both lipids are relatively minor lipids (less than 10% of total lipids) but have complex functions. The synthesis of both lipids requires the liponucleotide, CDP-DAG. CDP-DAG is synthesized from PA and CTP, and the reaction is catalyzed by enzymes that display CDP-DAG synthase activities. Despite sharing a common precursor, CDP-DAG, the synthesis of PI occurs at the endoplasmic reticulum (ER) whilst CL is synthesized in the mitochondria (Figure 1).

The importance of PI, in part, derives from its inositol ring as well as from its acyl chain composition. The inositol ring of PI can be phosphorylated at three positions, either singly or in combination, resulting in seven derivatives. Many of the phosphorylated PIs, including PI(4,5)P_2_, provide docking sites for reversible recruitment of proteins to membranes and to regulate protein function (Di Paolo and de Camilli, 2006; Balla, 2013; Choy et al., 2017; Raghu et al., 2019). Many transmembrane proteins, including ion channels and G-protein-coupled receptors, are known to be bound to PI(4,5)P_2_, which regulates their activity (Hille et al., 2015; Yen et al., 2018; Robinson et al., 2019). PI(4,5)P_2_ is also a substrate for two signaling enzymes, PLC, and PI3K (Vanhaesebroeck et al., 2012; Cockcroft and Raghu, 2016). PI(4)P has important roles in membrane trafficking (D’Angelo et al., 2008), as well as being utilized by many lipid transporters as a counter transport molecule. This allows movement of cholesterol or PS from the ER to other organelles, including the PM (Chung et al., 2015; Moser von Filseck et al., 2015). The acyl chain composition of PI and its phosphorylated derivatives are also unique. Most phospholipids contain a variety of acyl chains, varying in length as well as in the number of double bonds. Phosphoinositides are atypical; the main acyl chains present are C18:0 (stearic acid) at the *sn*-1 position and C20:4 (arachidonic acid) at the *sn*-2 position (Lee et al., 2012; Anderson et al., 2013; Naguib et al., 2015; Traynor-Kaplan et al., 2017; Mujalli et al., 2018; Barneda et al., 2019; Blunsom and Cockcroft, 2020). However, cultured cell-lines are more variable in their acyl chain composition (Naguib et al., 2015; Traynor-Kaplan et al., 2017). PI can attain its distinctive acyl chain composition during its synthesis and also through acyl chain remodeling after its synthesis (Imae et al., 2012; Lee et al., 2012; Anderson et al., 2013; Hishikawa et al., 2014).

Like PI, CL is unique compared to all other phospholipids, and it is the signature lipid of mitochondria. CL is essentially a lipid dimer with four acyl chains. The phosphate groups of two PA moieties are connected with a glycerol backbone to form a dimeric structure (Mejia et al., 2014). Like PI, the fatty acid profile of CL can also be quite specific. Tissues such as heart and skeletal muscle, require high mitochondrial metabolic activity and are greatly enriched in tetra-linoleoyl-CL ((C18:2)_4_-CL). The C18:2 acyl chains are acquired after its synthesis by acyl chain remodeling by tafazzin, a CoA-independent phospholipid acyltransferase (Schlame and Greenberg, 2017). Similar to PI and its derivatives, CL has a plethora of functions mainly confined to mitochondria. By associating with the major proteins of the mitochondrial respiratory chain, CL increases the efficiency of electron flow and ATP/ADP exchange. CL is also required for the electron transport chain super-complex formation, maintenance of cristae morphology, mitophagy and facilitating mitochondrial fission/fusion (Acehan et al., 2011; Dudek et al., 2013; Horvath and Daum, 2013; Baile et al., 2014; Dudek, 2017; Ikon and Ryan, 2017; Maguire et al., 2017; Schlame and Greenberg, 2017).

Synthesis of PA by acylation of glycerol-3-phosphate is the initiating event in phospholipid biosynthesis in both prokaryotic and eukaryotic organisms. The next step is the conversion of PA into CDP-DAG, the central liponucleotide intermediate for phospholipid biosynthesis. In bacteria, CDP-DAG is the precursor for the biosynthesis of all the major phospholipids including PG, CL, PS, and PE (produced through decarboxylation of PS) (López-Lara and Geiger, 2017). In eukaryotes, PA is a precursor for both CDP-DAG and DAG; CDP-DAG is used to make PI, PG, and CL, while DAG is required for PC, PE, and TAG synthesis (Yang et al., 2018). In yeast, PS is produced from serine and CDP-DAG (Henry et al., 2012).

The enzyme CDS (alternative name: CTP:phosphatidate cytidylyltransferase) catalyzes the synthesis of CDP-DAG from CTP and PA. CDS and its homologs were initially identified as integral membrane proteins responsible for the CDP-DAG synthase activity in bacterial PMs and the ER membranes of eukaryotic cells (Icho et al., 1985; Sparrow and Raetz, 1985; Kelley and Carman, 1987; Shen and Dowhan, 1997). In 1992, a study using rat liver discovered that mitochondria also contain an intrinsic CDP-DAG synthase activity separate from the ER-localized CDS activity (Mok et al., 1992). GTP stimulated the CDS activity in the microsomes (mainly ER) whilst the mitochondrial activity was insensitive to GTP. Twenty-five years later, in 2013, the CDP-DAG synthase activity of yeast mitochondria was found to be due to Tam41, a peripheral membrane protein present on the mitochondrial inner membrane (Tamura et al., 2013). Tam41 and CDS both catalyze the synthesis of CDP-DAG using PA and CTP as substrates, but share no sequence or structural homology (Liu X. et al., 2014; Jiao et al., 2019). CDS is an integral membrane protein, whilst Tam41 is a peripheral membrane protein that binds to the membrane through its C-terminal domain (Liu X. et al., 2014; Jiao et al., 2019).

In this review, we focus on the two unrelated families of enzymes that possess CDS activity, CDS/Cds and TAMM41/Tam41. In mammalian cells, PI synthesis is confined to the ER whilst PG and CL synthesis is restricted to the mitochondria. During PLC signaling, the rapid consumption of PI(4,5)P_2_ requires its replenishment and for this PI resynthesis is essential. The role of CDS enzymes in this process is discussed. We review recent evidence that suggest that CDS enzymes are regulated, and regulation is dependent on context. We examine how disruption of CDS enzymes can lead to metabolic disturbances, particularly, the formation of super-sized lipid droplets. CDS enzymes are expressed in protozoa and we discuss their spatial segregation from other enzymes required for PI and CL synthesis in other organelles. Finally, we suggest that the CDS family of enzymes may also contribute to CL synthesis in addition to TAMM41 requiring transfer of CDP-DAG from the ER membrane to mitochondrial membranes.

## Identification of Prokaryotic and Eukaryotic Cds Enzymes

**FIGURE 2:**
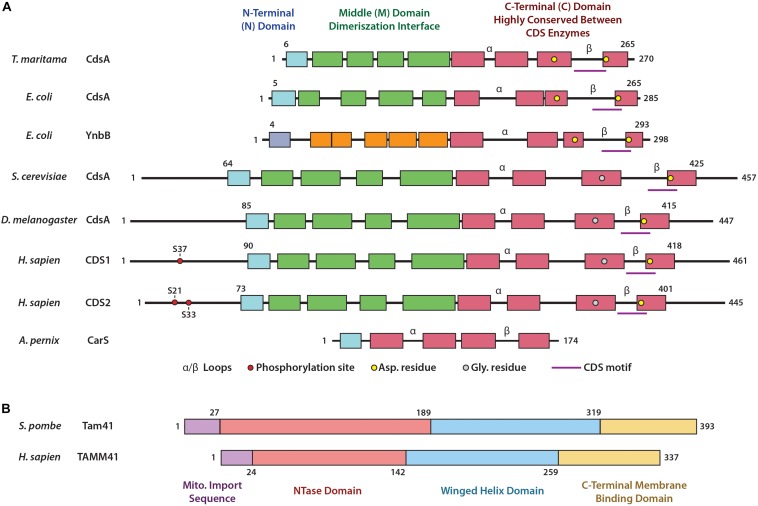
Domain structures of CDS enzymes and of Tam41. **(A)** Domain structures of the CDS enzymes based upon the bacterial enzyme *Tm*CdsA from *T. maritama*. These include the N-terminal domain (blue), the Middle domain involved in dimerization (green) and the highly conserved catalytic C-terminal domain (red). Within the C-terminal domain lies the α and β loops, the CDS motif, and residues involved in coordination of metal ions crucial for catalysis. Phosphorylation sites of *Hs*CDS1/2 are also included. **(B)** Domain structures of TAMM41 enzymes based upon the yeast enzyme *Sp*Tam41 from *S. pombe*: these include the mitochondrial import sequence (purple), the NTase domain (orange) and winged helix domain (blue) (which form the catalytic binding pocket), and the C-terminal membrane binding domain (yellow).

The gene encoding for *Cds* was first cloned in *Escherichia coli* and codes for a 27 kDa protein, predicted to contain transmembrane regions (Icho et al., 1985). The protein was purified and analysis of the acyl chain preference for PA indicated that the enzyme displays a striking preference for PAs bearing at least one double bond in their acyl moieties (Sparrow and Raetz, 1985). The first eukaryotic *Cds* was cloned from *Drosophila.* This gene encodes a polypeptide of 447 amino acids with a molecular weight of 49 kDa (Wu et al., 1995). *Drosophila* Cds shares a 31% amino acid identity with bacterial Cds. Subsequently, the Cds cDNA was cloned from the yeast, *Saccharomyces cerevisiae*; the cDNA encodes a protein of 457 amino acids with a molecular mass of 52 kDa and shares 37% identity and 60% similarity with the *Drosophila* enzyme (Shen et al., 1996). Importantly, these two eukaryotic enzymes possess a hydrophilic N-terminus, which is absent in the *E. coli* Cds enzyme (Figure 2A).

Cloning of CDS cDNA from human (Heacock et al., 1996; Lykidis et al., 1997; Weeks et al., 1997; Halford et al., 1998), mouse (Volta et al., 1999; Inglis-Broadgate et al., 2005), rat (Saito et al., 1997), and pig (Mercade et al., 2007) soon followed and two *CDS* genes encoding for CDS1 and CDS2 were identified. It is thought that they arose during a gene duplication event 500–900 million years ago. Thus, insects contain a single copy whilst fish and mammals possess two copies (Lykidis, 2007). Not surprisingly, all eukaryotic genomes, sequenced to date, contain Cds homologs (Lykidis, 2007). The number of *Cds* genes varies in different organisms. For example, *C. elegans* has one *Cds* gene, *Arabidopsis thaliana* genome has 5 *Cds* genes, whilst zebrafish has 2 genes. Analysis of *Cds* genes in the protozoan parasite *Toxoplasma gondii* revealed the presence of two phylogenetically divergent CDS enzymes, a eukaryotic and a prokaryotic type (Table 1). The CDS sequences fall into two discrete eukaryotic and prokaryotic clades (Kong et al., 2017) with the CDS signature motif (KDX_5_PGHGGX_2_DRXD; X being any amino acid) found in both clades. The CDS signature motif is present at the C-terminal region of the protein (Figure 2A). The prokaryotic-type sequences are found in bacteria, cyanobacteria, red algae and selected protozoa (e.g., *Leishmania major*, *Eimeria falciformis*, and *Trypanosoma cruzi*) but not others (e.g., *Cryptosporidium parvum*, *Plasmodium falciparum*, and *Trypanosoma brucei*) (Table 1; Kong et al., 2017).

**TABLE 1:** CDS enzymes in different organisms.

Species	Prokaryote-type CDS	Eukaryote-type CDS	Tamm41	References
Human, Mice, rat, pig, zebrafish	None	CDS1 and CDS2	TAMM41	Kelley and Carman, 1987; Blunsom et al., 2017
*S. cerevisiae* and *S. pombe* (yeast)	None	CdsA	Tam41	Tamura et al., 2013
*D. melanogaster* (Flies)	None	CdsA	Tam41	Wu et al., 1995
*A. thaliana* (plant)	*At*Cds4, *At*Cds5	*At*Cds1, *At*Cds2, *At*Cds3	Tam41	Zhou et al., 2013; Kong et al., 2017
*T. cruzi* (Kinetoplastid)	*Tc*Cds2	*Tc*Cds1	None	Kong et al., 2017
*T. brucei* (Kinetoplastid)	None	*Tb*Cds	None	Lilley et al., 2014
*E. falciformis* (Apicocomplexan)	*Ef*Cds2	*Ef*Cds1	None	Kong et al., 2017
*T. gondii* (Apicocomplexan)	*Tg*Cds2	*Tg*Cds1	None	Kong et al., 2017
*P. falciparum* (Apicocomplexan)	None	*Pf*Cds	None	Kong et al., 2017
*E. coli* (Bacteria)	*Ec*CdsA, *Ec*YnbB,	None	None	Icho et al., 1985; Sato et al., 2019; Sawasato et al., 2019
*Synechocystis* sp. PCC6803 (cyanobacteria)	*Ss*Cds	None	None	Sato et al., 2000

**FIGURE 3:**
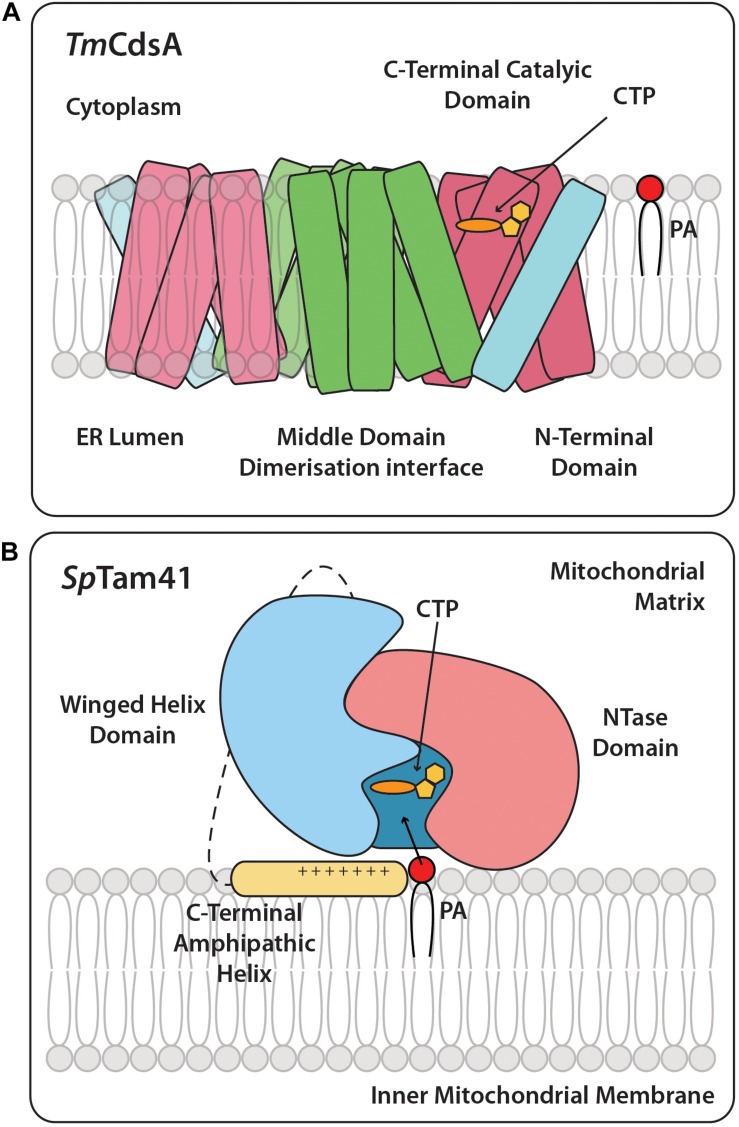
Cartoon structures of *Tm*CdsA and *Sp*Tam41. **(A)** CDS is an integral membrane protein and is present as a dimer and accepts the two substrates, CTP, and PA. On the cytoplasmic side of each *Tm*CdsA monomer, a funnel-shaped cavity indents half way into the membrane region. The cavity has two wide openings, which enable it to receive dual substrates, CTP from the cytoplasm and PA from the lipid bilayer at the same time. The conversion of CTP and PA into CDP-DAG and pyrophosphate occurs through a process involving the transfer of CMP group from CTP onto the phosphate group of PA. Adapted from Liu X. et al. (2014). Color coding is the same as *Tm*CdsA in Figure 2. **(B)** The full length *Sp*Tam41 exists as a monomer and is associated to the matrix side of the inner mitochondrial membrane by the membrane binding domain at the C-terminal region (colored yellow). CTP and PA sequentially bind to the active site of Tam41. The enzymatic conversion of CTP and PA into CDP-DAG and pyrophosphate occurs through a process involving the transfer of CMP group from CTP onto the phosphate group of PA. Adapted from Jiao et al. (2019). Color coding is the same as *Sp*Tam41 in Figure 2.

The structure of the bacterial *Thermotoga maritima* Cds enzyme, *Tm*CdsA, informs us that it is a dimer. Each of the *Tm*CdsA monomers contain nine transmembrane helices arranged in a novel fold containing three domains; the N-terminal domain containing a single helix (colored blue), the middle dimerization domain containing 4 helices (colored green), and the C-terminal domain comprising of four helices that contains the catalytic site (colored red) (Liu X. et al., 2014; Figures 2A, 3A). Within each monomer, a funnel-shaped cavity forms penetrating halfway into the membrane. This cavity is shaped by amino acid residues from the C-terminal domain and the N-terminal domain. The cavity has dual openings allowing the simultaneous acceptance of the hydrophilic CTP and the hydrophobic PA substrate. At the bottom of the cavity, a dyad forms between ^219^Asp and ^249^Asp that coordinates a magnesium-potassium hetero-di-metal center. The position of the asparagine residues is shown in Figure 2A as yellow circles. Only ^249^Asp residue is conserved in all CDS sequences. ^219^Asp is only conserved in prokaryotes and is replaced by a glycine residue (Figure 2A; gray circles) in eukaryotes that presumably also coordinates the Mg^2+^. The dyad is key to the catalysis of CDP-DAG formation. The implied mechanism of this reaction is that the Mg^2+^ ion activates the phosphate head group of PA, permitting nucleophilic attack on the α-phosphate of CTP. Simultaneously, the K^+^ ion binds the remaining β and γ phosphates of the CTP, and facilitates release of the products, CDP-DAG, and pyrophosphate from the active site. Two conserved cytoplasmic loops, α and β, surround the active site and contribute to substrate binding (Figure 2A).

The three domains of life are Archaea, Bacteria and Eukaryota and they differ in their phospholipids. Whilst phospholipids of bacteria and eukaryotes consist of linear acyl chains ester-linked or ether-linked to glycerol-3-phosphate, archaeal phospholipids consist of isoprenoid chains ether-bonded to glycerol-1-phosphate (Lombard et al., 2012). Nonetheless, the key step in phospholipid synthesis is the same in all three domains of life: the transfer of CMP from CTP to a glycerol-phosphate backbone catalyzed by transmembrane enzymes. CDS uses PA whilst the CDP-archaeol synthase (CarS) uses DGGGP to make CDP-archeol (Jain et al., 2014). The structure of the CarS of *Aeropyrum pernix* (*Ap*CarS) comprises of five transmembrane helices and cytoplasmic loops which form a large charged cavity for CTP and the lipophilic substrate, DGGGP to bind (Ren et al., 2017). Part of the cavity is formed by two cytoplasmic loops, also contributing to substrate binding similar to *Tm*CdsA (Figure 2A). Unlike, the bacterial enzyme, *Ap*CarS lacks the dimerization domain. Nonetheless, *Ap*CarS is a structural homolog of *Tm*CdsA, although the two proteins only share 12% sequence identity. Thus, bacterial and archaeal transmembrane CTP transferases have likely evolved from a common ancestral enzyme.

## Mammalian CDS1 and CDS2

In humans, the genes for *Cds1* and *Cds2* are located on chromosome 4q21 and 20p13, respectively (Halford et al., 1998, 2002). The protein sequences of CDS1 and CDS2 show 72% identity and 92% similarity and the major difference is found at the N-terminal region (Halford et al., 1998; Inglis-Broadgate et al., 2005), where the phosphorylation sites are also found (Figure 2A). Human CDS1 is 461 amino acids long whereas CDS2 is slightly shorter at 445 amino acids due to a shorter N-terminal region prior to the first transmembrane domain. The mammalian CDS1 and CDS2 are integral membrane proteins which can form homodimers (Blunsom et al., 2017). Likewise, the bacterial CDS enzyme, *Tm*CdsA, from *T. maritama* is also a dimer (Figure 3A; Liu X. et al., 2014). The purification of the Cds enzyme from yeast also identified a homodimer of two identical 56 kDa subunits (Kelley and Carman, 1987; Carman and Kelley, 1992). Furthermore, it is notable that in the BioGRID database^1^, CDS1, and CDS2 interact with each other, suggesting that heterodimers may be possible. CDS enzymes are phosphorylated enzymes but whether this modification has any effect on enzyme activity remains to be studied. Using high throughput analysis, CDS1 was found to be phosphorylated at Ser37, conserved between human, mouse, and rat. CDS1 is also ubiquitylated at Lys270, which is also conserved across species. In human CDS2, Ser21 and Ser33 are phosphorylated whilst Lys253 and Lys328 are ubiquitylated (Figure 2A). Again, these modifications are conserved in both mice and rats (data mined from https://www.phosphosite.org).

The ER is a continuous membrane system comprising of the nuclear envelope, flat sheets at the central perinuclear region and a network of highly curved tubules at the periphery. The subcellular localization of mammalian CDS1 and CDS2 enzymes has been mainly studied by over-expression of tagged proteins in several different cell types including COS-7 and CHO-1 cells, and the consensus is that both enzymes localize to the ER membranes (Saito et al., 1997; Inglis-Broadgate et al., 2005; Kim et al., 2011; D’Souza et al., 2014). In COS-7 cells, both CDS1 and CDS2 are found at the nuclear envelope and the peripheral ER tubules. In contrast to CDS enzymes, PI synthase (PIS) localizes to the central perinuclear ER and peripheral ER tubules as well as at uncharacterized mobile structures in COS-7 cells (Kim et al., 2011). A recent study has identified that Cds1 is also at the inner nuclear membrane in yeast and contributes to lipid droplet formation in the nucleus (Romanauska and Kohler, 2018).

PI is highly enriched in C18:0 at the *sn-*1 position and C20:4 at the *sn-*2 position, and this enrichment could be due to the acyl chain specificity of PA utilized by CDS enzymes. Analysis of CDS1 and CDS2 using PA with different acyl chain compositions in a detergent/phospholipid/mixed micelle-based assay indicate that the two human isoforms show different acyl chain specificities. CDS2 is selective for the acyl chains at the *sn-*1 and *sn-*2 positions, the most preferred species being *sn-*1-stearoyl, *sn-2*-arachidonoyl PA (C18:0/20:4-PA). In contrast, CDS1 showed no particular substrate selectivity, displaying similar activities for almost all substrates tested (D’Souza et al., 2014). However, in a different study, rat CDS1 showed the highest *in vitro* activity when C18:0/C20:4-PA was used as substrate with almost no activity detected toward PA containing saturated fatty acyl groups in both of the *sn*-1 and *sn*-2 positions (Saito et al., 1997). Although, rat CDS1 was found to prefer C18:0/C20:4-PA as a substrate, both PA from egg yolk lecithin and di-C18:1-PA gave reasonable activity (Saito et al., 1997). As these assays are done *in vitro* in the presence of detergent, the selectivity for PA species remains an open question. The important point to emphasize is that CDS1 is non-selective for the PA species and will equally utilize C18:0/20:4-PA as substrate. Additional work with purified enzymes and *in vitro* assays without detergent are required to clarify this question. It is notable that PIS that converts CDP-DAG to PI appears to show no selectivity for the acyl chain composition (D’Souza and Epand, 2015). The identification of PI remodeling enzymes suggests that a major route to the enrichment of PI with C18:0 and C20:4 probably occurs subsequent to its synthesis (Barneda et al., 2019; Blunsom and Cockcroft, 2020).

## Functional Analysis of CDS Enzymes During Phospholipase C Activation

The formation of CDP-DAG is the rate limiting step in the synthesis of PI. When PLC is activated for prolonged periods, as much at 30–40% of the total cellular PI is depleted (Blunsom et al., 2019), and therefore the extent of CDS activity would determine the rate of PI resynthesis. Studies in two model organisms, *Drosophila* and zebrafish, suggest that CDS activity controls both the availability and the extent of PI(4,5)P_2_-dependent signaling. Mutation of the single *Cds* gene in *Drosophila* leads to light-induced irreversible loss of phototransduction and retinal degeneration (Takeishi et al., 2007; Dudek et al., 2013; Zhao et al., 2019). In zebrafish, CDS-dependent phosphoinositide availability limits VEGFA signaling (Baile et al., 2014).

The importance of CDS enzymes comes from the need for the cells to maintain their phosphoinositide levels, in particular, PI(4,5)P_2_. PI(4,5)P_2_ is a substrate for PLC and for PI3K. Both enzymes are activated when agonists, hormones and neurotransmitters interact with their cell surface receptor. Activation of PI3K converts PI(4,5)P_2_ to PI(3,4,5)P_3_; although the amount of PI(3,4,5)P_3_ produced is very small compared to the resting pool of PI(4,5)P_2_, PI3K signaling is disturbed when PI(4,5)P_2_ resynthesis is disrupted as discussed below.

**FIGURE 4:**
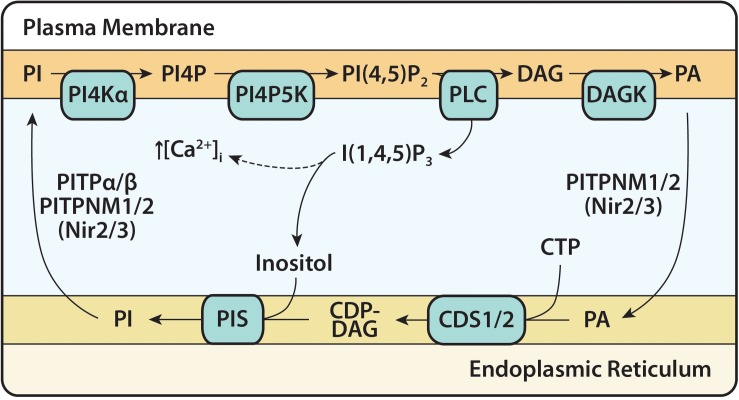
The PI(4,5)P_2_ cycle. The PI(4,5)P_2_ cycle begins with the hydrolysis of PI(4,5)P_2_ and formation of the second messengers, I(1,4,5)P_3_ and DAG. I(1,4,5)P_3_ causes an increase in intracellular calcium concentration before being dephosphorylated into inositol. DAG is phosphorylated to PA at the plasma membrane (PM) by DAG kinase (DAGK) and transferred to the ER via Class IIa PITPs (PI/PA transfer proteins) PITPNM1/2. At the ER, PA, and CTP are converted to CDP-DAG by CDS enzymes (CDS1 and CDS2). CDP-DAG is synthesized into PI and this is catalyzed by the enzyme PI synthase (PIS). PI is transferred to the PM by Class I PITPs (PI/PC transfer proteins) PITPα/β and Class IIa PITPNM1/2, for phosphorylation to PI(4,5)P_2_ by the resident enzymes, PI4KIIIα and PIP5K. PITPNM1/2 are also known as Nir2/Nir3.

The PI(4,5)P_2_ cycle starts with the hydrolysis of PI(4,5)P_2_ by PLC at the PM (Figure 4). This forms the second messengers inositol triphosphate [I(1,4,5)P_3_] and DAG. The soluble I(1,4,5)P_3_ causes the release of intracellular calcium ions from the ER lumen; I(1,4,5)P_3_ gets dephosphorylated to inositol where it is re-used for PI synthesis. The membrane-bound DAG activates PKC; DAG is removed by phosphorylation to PA by DAG kinases (DAGK). The newly formed PA is transported to the ER by Class IIa PI/PA transfer proteins (PITPNM1/2 (alt. name Nir2/3)) (Garner et al., 2012; Cockcroft and Raghu, 2016). At the ER, PA is converted into CDP-DAG by CDS enzymes, the rate-limiting step in the synthesis of PI (MacDonald et al., 1975; Nakagawa et al., 1989) and in the PI(4,5)P_2_ cycle, CDP-DAG is then converted into PI by PIS. Once PI is formed, either Class I PI/PC transfer proteins (PITPα/β) or Class IIa PITPNM proteins can transport it to the PM where it undergoes two sequential phosphorylations by resident lipid kinases to regenerate PI(4,5)P_2_. This completes the PI(4,5)P_2_ cycle (Figure 4).

During PLC signaling, the PA formed at the PM will be enriched with stearic acid (C18:0) and arachidonic acid (C20:4) reflecting the PI(4,5)P_2_ composition (Bozelli and Epand, 2019a; Blunsom and Cockcroft, 2020). Similarly, when PLD hydrolyzes PC, which usually occurs concurrently with PLC activation, the resultant PA reflects the fatty acid composition of PC (Pettitt et al., 1997). The *sn-*1 position of PC is mainly C16:0 and C18:0 and the *sn-*2 position is mainly C18:1 and C18:2. Whether these different PA species are kept metabolically separate at the PM is not known. Since the PI(4,5)P_2_ cycle occurs at membrane contact sites, the intermediates of the cycle may remain compartmentalized (Cockcroft and Raghu, 2016). It has been generally assumed that the PA produced due to PLC activation remains in the PI(4,5)P_2_ cycle and is re-used for PI resynthesis thus maintaining the fatty acid composition (Michell, 1975; Broekman et al., 1981). However, in a study of the PI acyl chain composition of platelets, the fatty acid profile of PI had changed after stimulation with thrombin. The enrichment of C18:0/C20:4 was no longer apparent, implying newly formed PI, made through *de novo* synthesis, lacked any specificity to create the typical profile of PI (Prescott and Majerus, 1981).

To maintain the C18:0/C20:4 of PI during the PLC-PI(4,5)P_2_, other enzymes in the cycle such as DAGK would also need to show selectivity. There are 10 mammalian DAGK subdivided into five types and are differentially expressed. DAGKε is the only enzyme that shows selectivity for C18:0/C20:4-DAG *in vitro* and has been suggested as the DAGK responsible for metabolizing PLC-derived DAG (Lung et al., 2009; Shulga et al., 2011; Epand et al., 2016; Sakane et al., 2018). The enzyme localizes to the ER raising the question of its role in phosphorylating DAG at the PM during the PI(4,5)P_2_ cycle (Nakano et al., 2016). EM immuno-tomography has shown that DAGKε localizes at ER-PM membrane contact sites in Purkinje cells in the brain (Hozumi et al., 2017). Thus, it is suggested that DAGKε can access DAG from the PM and deliver it to the ER (Bozelli and Epand, 2019a). However, the enzyme is not ubiquitously expressed indicating that depending on cell-type, different DAGKs could be more relevant. Another DAGKα, an isoform enriched in T lymphocytes, is cytosolic and translocates to the PM upon stimulation suggesting that this DAGK could be responsible for DAG phosphorylation in some cells including T cells (Sanjuan et al., 2001; Mérida et al., 2015). It is non-specific for the substrate meaning that C18:0/C20:4-DAG could be utilized as well (Mérida et al., 2015).

The PI(4,5)P_2_ cycle is not a closed cycle. The intermediates of the PI(4,5)P_2_ cycle such as DAG and PI can exit the cycle and are not necessarily recycled back to produce more PI(4,5)P_2_ (Rohit et al., 2018). Both DAG and PI can be metabolized and exit the PI(4,5)P_2_ cycle; DAG is degraded by DAG lipase to the endocannabinoid, 2-arachidonoylglycerol (2-AG) and PI to *sn*-2-arachidonoyl lyso-PI by the phospholipase A1, DDHD1 (Inloes et al., 2018). Both are bioactive metabolites acting as agonists on cell surface receptors (Alhouayek et al., 2018; Baggelaar et al., 2018). Thus newly synthesized PI has to enter the PI(4,5)P_2_ cycle to maintain phosphoinositide levels. The question of how *de novo*-synthesized PI becomes enriched with its characteristic fatty acid composition is likely through the remodeling of PI by phospholipase A and PI acyl transferases (Barneda et al., 2019; Bozelli and Epand, 2019b; Blunsom and Cockcroft, 2020). As per the other phospholipids, PI has its own set of acyl transferases, LPIAT1 (also known as MBOAT7) and LYCAT (also known as AGPAT8), which allow a cycle of deacylation-reacylation to take place, enriching PI with specific acyl chains. LYCAT adds stearic acid (C18:0) to the *sn*-1 position and LPIAT1 adds arachidonic acid (C20:4) to the *sn*-2 position.

Studies in *Drosophila*, zebrafish and cultured mammalian cell-lines all highlight the importance of CDS enzymes in phosphoinositide signaling. All these studies suggest that CDS activity controls the extent of PI(4,5)P_2_-dependent signaling. Vertebrates have two CDS genes, *Cds1* and *Cds2*, and the protein products of both genes localize to the ER (D’Souza et al., 2014). Of the two CDS enzymes, is there a separation of function for *de novo* synthesis of PI versus resynthesis of PI during PLC signaling? CDS1 has a restricted level of expression whilst CDS2 expression is ubiquitous (Saito et al., 1997; Volta et al., 1999; Inglis-Broadgate et al., 2005). As described later, *Cds* genes can also be induced when high rates of PI synthesis are required (Blunsom et al., 2019).

Studies from *Drosophila* highlight the importance of CDS in the visual phototransduction system. The underlying phototransduction machinery in photoreceptor neurons is rhodopsin-based Gαq-protein-coupled signaling cascade where light activates PLCβ, encoded by the *norpA* gene. Disruption of the *Cds* gene in photoreceptor cells limits PI(4,5)P_2_ availability and therefore PLC-mediated signaling leading to a decreased amplitude of the light response (Wu et al., 1995; Hardie et al., 2015; Liu et al., 2018). In the fly there is a single *Cds* gene but with two transcripts (Wu et al., 1995). Loss of the *Cds* gene is lethal. Mutations in the eye-specific *Cds* gene results in a defect in phototransduction and retinal degeneration. Light activation results in PI(4,5)P_2_ hydrolysis that cannot be replenished in the *eye-Cds* mutants. Thus, *Cds* mutants cannot sustain a light-activated current. Interestingly, the Cds enzyme was rate-limiting as transgenic animals that over-expressed the enzyme 4–5 fold display a large and significant increase in the amplitude of the light response when compared to wild-type animals (Wu et al., 1995).

In zebrafish, vascular growth is dependent on VEGFA-stimulated PLC signaling and is reliant on CDS enzymes for PI(4,5)P_2_ availability (Pan et al., 2012). Like other vertebrates, zebrafish also have two CDS genes, *Cds1* and *Cds2* but only *Cds2* mutants result in vascular-specific defects *in vivo.* Vascular growth is mainly governed by angiogenesis where new blood vessels are formed from existing vessels, whilst vasculogenesis is the *de novo* formation of blood vessels using stem cells. In addition, vessel regression also plays important roles in embryonic development and tissue homeostasis. VEGFA, acting through the tyrosine kinase receptor, VEGF receptor 2 on endothelial cells, governs vasculogenesis, angiogenesis and regression and acts through the activation of both PLCγ and PI3K. Both use PI(4,5)P_2_ as their substrates. PI3K catalyzes the conversion of PI(4,5)P_2_ to PI(3,4,5)P_3_ and PLCγ hydrolyzes PI(4,5)P_2_. Using the zebrafish model system, mutations in *Cds2* resulted in vascular-specific defects *in vivo* and failure of VEGFA-induced angiogenesis *in vitro* (Pan et al., 2012; Hill et al., 2013). Interestingly, morpholinos that targeted CDS1 also caused a mild vascular phenotype and morpholinos that targeted both CDS1 and CDS2 resulted in early embryonic lethality. The CDS2 deficient phenotypes could be rescued by artificial elevation of PI(4,5)P_2_ and increased CDS2 levels promoted excess angiogenesis (Pan et al., 2012). As expected, CDS2 is enriched in the endothelium (Pan et al., 2012).

In addition to vascular defects, in zebrafish, CDS2 mutants switched the output of VEGFA signaling from promoting angiogenesis to inducing vessel regression (Zhao et al., 2019). It was suggested that without the CDS2-controlled PI(4,5)P_2_ resynthesis, the VEGFA-PLCγ signaling axis hydrolyzes PI(4,5)P_2_ leading to depletion of PI(4,5)P_2_ and thus inhibition of PI3K-catalyzed PI(3,4,5)P_3_ formation. FOXO1 accumulation in the nucleus can thus trigger reverse migration of angiogenic endothelium. Live cell imaging of zebrafish revealed the reverse migration of the angiogenic endothelium in the CDS2 mutant upon VEGFA stimulation.

The observations made in zebrafish were also made in mice. *CDS2* was deleted specifically in the adult endothelium in mice and endothelial regression was observed in the postnatal retina. Furthermore, in tumor models, endothelial CDS2 deficiency induced vessel regression, and suppressing tumor growth. Stimulation by VEGFA reduced PI(4,5)P_2_ which could not be replaced in the absence of CDS2-controlled PI synthesis. This caused a deficiency in PI(3,4,5)P_3_ permitting FOXO1 to trigger regression of CDS-null endothelium (Zhao et al., 2019). These results confirm the importance of CDS2 in maintaining PI(4,5)P_2_ levels during PLC signaling. In its absence, it causes disturbances in PI(3,4,5)P_3_ as well.

In cardiac myocytes, the Gαq-protein-coupled receptor agonists such as angiotensin II, vasopressin, endothelin-1, and phenylephrine activate PLC. Cardiac myocytes respond to physiological and pathological stimuli by increasing their size (hypertrophy) and Gαq-phosphoinositide signaling system is responsible for the development of pathological hypertrophy (Arimoto et al., 2006; Takeishi et al., 2007; Niizeki et al., 2008; Maillet et al., 2013). Sustained vasopressin signaling in a H9c2 cardiac cell line over a 24 h period recapitulates the hypertrophic response and this is coupled to continuous PLC signaling (Blunsom et al., 2019). PI levels are depleted by 50% under these conditions, and the cells respond by upregulating the expression of CDS1. CDS2 expression is unaffected. The increase in CDS1 mRNA was dependent on PKC activation of cFos expression (Blunsom et al., 2019). Other studies using cardiomyocytes have reported that other agonists such as norepinephrine also stimulate cFos expression due to PLC activity via PKC likely increasing CDS1 expression as well (Singal et al., 2009).

Interestingly, cFos is not only a regulator of CDS1 mRNA expression, it can also directly bind to CDS1 at the ER and activate it (Alfonso Pecchio et al., 2011; Cardozo Gizzi and Caputto, 2013). The N-terminal domain of cFos interacts with CDS1 resulting in the increase in CDS1 activity *in vitro* and also in tumor cells. Growth of tumor cells require high rates of lipid synthesis to support membrane biogenesis, and two transcription factors of the AP-1 family that activate phospholipid synthesis are Fra-1 and cFos. In breast tumors, both transcription factors are also expressed in the cytoplasm as well as the nucleus (Motrich et al., 2013). For cFos and Fra-1 to promote activation of phospholipid synthesis, it associates with CDS1 in the ER through the cFos N-terminal domain (1–38 a.a.). The increase in the catalytic activity of CDS1 is through the basic domain (a.a. 139–159) of cFos. Using FRET analysis, the N-terminus of Fra-1 and cFos was shown to physically associate with CDS1 in cells. Interestingly, expression of the N-terminus of either cFos or Fra-1 resulted in a decrease in the proportion of full-length Fra-1 or cFos at the ER compartment. Expression of the N-terminal domain of Fra-1 or cFos in MDA-MB231 or 4T1 cells results in inhibition of cell growth, both in tissue culture and when examined in a Balb/c mice tumor model. When the tumor was excised from the mice, and the CDS activity monitored, an increase was observed that could be reversed upon stripping the tumor samples with IM KCl to remove the Fra-1 and cFos from the membranes (Racca et al., 2019).

To summarize, there is now substantial evidence that PI synthesis following PLC activation is dependent on CDS enzymes. In the case of the zebrafish, CDS2 appears to be more important than CDS1, whilst in the cardiomyoblast cell line H9c2, CDS1 appears to be more important. Importantly, the level of CDS expression appears to regulate the extent of signaling.

## CDS Enzymes Determine Size of Lipid Droplets

Phosphatidic acid is a substrate for CDS enzymes, as well as a substrate for lipins. Thus, PA can be channeled to make CDP-DAG or used to make DAG. DAG can then be routed to either TAG or to the synthesis of PC and PE. Inhibition of CDS enzymes would result in an increase in PA, which could be redirected to generate DAG by lipins. DAG could then be channeled to TAG or to PC and PE synthesis. Loss-of-function CDS mutants have been identified in both budding and fission yeast, *Drosophila* and in cultured mammalian cells and in all cases, results in the formation of super-sized lipid droplets (LDs) (Fei et al., 2011; He et al., 2014; Liu Y. et al., 2014; Qi et al., 2016).

Lipid droplets are highly dynamic organelles that originate from the ER before they expand and mature. Super-sized LDs can form either by expansion or by coalescence of contacting LDs. Studies in both budding and fission yeast show that loss of function mutations in *Cds1* lead to enlarged lipid droplets. However, the mechanism appears to be different. In the budding yeast *S. cerevisiae*, PA has been identified as one of the regulators of LD growth. In a genome-wide screen, ten yeast mutants producing super-sized LDs were isolated. The mutated genes included *Cds1*, and biochemical and genetic analyses revealed that a common feature of the mutants was an increase in PA. PA was found to facilitate the coalescence of contacting LDs, resulting in the formation of super-sized LDs (Fei et al., 2011; Qi et al., 2016). In contrast, in the fission yeast, *S. pombe*, no increase in PA was observed but an increase in DAG and TAG was found, suggesting that cellular PA was shunted toward TAG biosynthesis. The LDs were in close proximity to the ER and it was suggested that growth of the LDs was by expansion ascribed to the presence to TAG synthases on the LDs (He et al., 2014).

In cultured HeLa cells (human) and 3T3-L1 preadipocytes (murine), downregulation of either CDS1 or CDS2 by RNAi resulted in super-sized LDs but only in the presence of exogenously added oleic acid (C18:1) (Qi et al., 2016; Xu et al., 2019). Addition of oleic acid to untreated cells led to an increase in TAG levels and this was marginally increased in CDS2 siRNA-treated cells. The formation of super-sized LDs appears to be dependent on the modulation of PA levels at the ER and on the lipid droplets, similar to that seen in *S. cerevisiae* (Xu et al., 2019). Knockdown of Lipin1 also resulted in super-sized LDs and this could be reversed by over-expression of either CDS1 or CDS2. Importantly, knockdown of Lipin1 did not increase TAG levels but only increased PA levels, confirming that the increase in PA was likely responsible for the super-sized lipid droplets. Overexpression of CDS1 or CDS2 decreased PA levels and inhibited lipid droplet expansion. Thus, this data supports the model that the super-sized LD phenotype is partly due to changes in PA and not just due to increased TAG synthesis.

Although knockdown of either CDS1 or CDS2 resulted in super-sized lipid droplets, the mechanisms appear to be different (Xu et al., 2019). LD size in CDS1-deficient cells could be restored by downregulating Fsp27/CIDEC (cell death-inducing DFF45-like effector C). CIDEC is a LD-associated protein that promotes lipid droplet growth by TAG transfer from smaller to larger LDs (Gong et al., 2011); in CDS1-knockout cells, CIDEC levels were increased significantly. Conversely, knockdown of CIDEC restored LD size to normal levels in the CDS1-knockout cells. This confirmed that super-sized LD formation in CDS1-deficient cells results from enhanced LD fusion/lipid transfer mediated by CIDEC proteins (Barneda et al., 2015). On the other hand, reducing expression of DGAT2 and GPAT4 rescued the enlarged LD-phenotype in CDS2-deficient cells. CDS2 deficiency promoted the LD association of DGAT2 and GPAT4. GPAT4 produces PA whilst DGAT2 consumes PA to make TAG. Thus, increased TAG synthesis could be responsible for super-sized LD formation.

Besides a potential role of PA in LD expansion, PA is also implicated in the differentiation of adipocytes. PA accumulation in the ER membrane upon CDS1/CDS2 depletion is thought to inhibit adipocyte differentiation by possibly interfering with PPARγ function required for the differentiation process. Depletion of CDS1 by siRNA resulted in inhibition of adipocyte differentiation, whereas depletion of CDS2 had a moderate inhibitory effect. It is notable that knockdown of CDS2 results in an increase in CDS1 mRNA (Qi et al., 2016).

## Down-Regulation of CDS Enzymes Causes Disturbances in PI Levels

In HeLa cells, knockdown of CDS1 or CDS2 decreased PI levels, whilst knockdown of CDS2 also led to a decrease in PG (Qi et al., 2016). The decrease in PI levels was not dependent on the presence of oleic acid, unlike the super-sized lipid droplets. In H9c2 cells, a decrease in all phosphoinositides was observed: the decrease was approximately 30% for PI, PIP and PIP_2_ in either CDS1 or CDS2 knockdown cells. No effects of CDS1 or CDS2 knockdown on CL was observed further supporting the findings that TAMM41 is responsible for CL synthesis.

Phosphoinositides have important functions in cells including maintaining the cytoskeletal network. Upon knockdown of CDS1 and CDS2 by siRNA, H9c2 cell morphology was drastically altered (Blunsom et al., 2019). Although the knockdown of CDS1 and CDS2 led to similar decreases in phosphoinositide levels, there were subtle differences in the morphological changes. The stress fibers usually seen in the H9c2 cells had lost form and become wispy; the F-actin strands were depolymerized, and a lot of the actin had begun to pool around the edges of the cells. This was much more pronounced in the CDS-knockdown cells. The knockdown cells were significantly smaller than the untreated cells, with CDS2 siRNA-treated cells being much more affected. This phenotype is similar to that observed in *Drosophila* salivary glands discussed below (Liu Y. et al., 2014). Also, the mitochondria and the trans golgi network (TGN) became fragmented. These drastic changes observed clearly indicate that disruption in the levels of phosphoinositides has a pleiotropic impact on the cells.

## Functional Studies of CdsA Requirement in Different Tissues in *Drosophila*

Several studies undertaken in *Drosophila* have examined *CdsA* function in specific tissues at different stages of development and in adult flies. This provides a snapshot of how an enzyme participates in functional responses in the context of the specific cellular proteome. Tissue-specific phenotypes are observed when the *CdsA* gene is disrupted in particular tissues. Deletion of the *CdsA* gene in the whole organism is lethal but the use of RNAi knockdown in specific cell types and tissues has provided a wealth of information about the specific impact of *CdsA*. *Drosophila* have a single *CdsA* gene, which has two transcripts. As the sole *Drosophila* CDS, *CdsA* is essential for embryogenesis (Liu Y. et al., 2014), phototransduction (Wu et al., 1995), metamorphosis (Liu et al., 2019), and spermatogenesis (Laurinyecz et al., 2016). Loss of *CdsA* perturbs lipid composition, including increased levels of PA coupled with the reduction of PG, PI, and its derivatives. Loss of *CdsA* function causes a variety of phenotypes in different tissues, which can be attributed to the alterations of one or more of these lipids.

The requirement of eye-specific *CdsA* in PLC-dependent phototransduction has already been described above where CdsA is required for maintaining phosphoinositide levels for PLC signaling. CdsA also plays important roles during development (Liu Y. et al., 2014). There is an increase of nearly 200-fold in body mass during the first 4 days of larval growth of *Drosophila*. Tissues including brain, salivary gland and imaginal discs do not accumulate neutral fat unlike the fat body at this stage. CdsA is widely expressed in larval tissues including salivary gland, fat body, and brain and muscle. Whole animal *CdsA RNAi*, which decreases CdsA transcripts by 40%, causes an increase in lipid accumulation due to an increase in TAG in many tissues including larval imaginal discs, brain, salivary gland, prothoracic gland, proventriculus, and hindgut. The most prominent response was in salivary glands and prothoracic gland. In addition to the fat storage phenotype, the cell size was much smaller. Analysis of the salivary gland indicated that loss of *CdsA* also resulted in a reduction in both PI(3,4,5)P_3_ and in phospho-Akt, downstream products of insulin stimulation. The total level of PI and PG were also reduced with an increase in PA. In contrast, overexpression of CdsA in the salivary gland resulted in an increase in both PI and PG. PI levels were also decreased when PIS transcripts were reduced. Similarly to *CdsA RNAi* salivary glands, *Pis RNAi* salivary glands were small and accumulated lipid droplets. In this case, the decrease in PI was accompanied by an increase in PG indicating that CDP-DAG was shunted toward PG synthesis. Thus, CdsA knockdown results in a decrease in PI and this ultimately disrupts insulin signaling which is critical for providing the growth signal. In addition, PA was diverted to make TAG resulting in ectopic lipid storage.

Interestingly, perturbation of the insulin pathway caused a reduction of CdsA transcripts (Table 2). In contrast, when FOXO1, a downstream target of phospho-Akt was deleted, CdsA levels increased. Thus it would appear that insulin signaling and CdsA modulate the balance of growth and lipid storage via a positive feedback loop. CdsA positively regulates the activity of the insulin pathway by provision of PI for PI(3,4,5)P_3_ production, and the insulin pathway increases the transcription of CdsA. Thus, this study conducted *in vivo* reveals the intrinsic connections between CDS, phosphoinositide metabolism and insulin signaling in coordinating cell growth and neutral lipid storage (Liu Y. et al., 2014).

**TABLE 2:** Regulation of CDS1 and CDS2 mRNA by different mechanisms.

Condition	Up-regulated	Down-regulated	References
Perturbation of the insulin pathway in *Drosophila* by Akt RNAi or by dominant-negative PI3K		CdsA	Liu Y. et al., 2014
Akt over-expression or by loss of Foxo in *Drosophila*	CdsA		Liu Y. et al., 2014
H9c2 cells stimulated with either vasopressin (16h) or with PMA (24 h)	CDS1		Blunsom et al., 2019
PGC-1α/β heart-specific knockout mice		CDS1	Lai et al., 2014
PGC-1α or PGC-1β over-expression in neonatal rat cardiac myocytes	CDS1		Lai et al., 2014
Palmitic acid: increases p53 and SIRT6. Both form a complex and bind to the promoters of CDS1 and CDS2	CDS1 and CDS2		Li et al., 2018
Loss of miR-16		CDS2	Yan et al., 2013
Differentiation of 3T3-L1-preadipocytes *in vitro* for 8 days	CDS1		Qi et al., 2016
Low ZEB1/2 expression in non-small cell lung cancer (NSCLC) cell lines	CDS1		Gemmill et al., 2011
ZEB1/2 over-expression H358 cell line		CDS1	Gemmill et al., 2011
AMPKa2^–/–^mice: CL levels decreased by 25% in heart and the mitochondria are disorganized		CDS2	Athea et al., 2007
*Tamm41* knockout in zebrafish causes defects in mitophagy	CDS1		Yang et al., 2019

Larvae metamorphosis to pupae after day 4 is disrupted in *CdsA RNAi* flies. Metamorphosis is dependent on the steroid hormone, ecdysone. Ecdysone is stored in the prothoracic gland and during larval to pupal transition, neuronal prothoracicotropic hormone triggers its release. Unlike the salivary gland where *CdsA RNAi* decreases the cell size, the cell size of the prothoracic gland is not reduced. Nonetheless, *CdsA RNAi* significantly reduces the level of PI(4,5)P_2_ (as well as PI), the substrate for PLC of the prothoracic gland. It is suggested that *CdsA RNAi*, by reducing the levels of PI(4,5)P_2_, subsequently leads to the reduction of PLC activation and thus I(1,4,5)P_3_/Ca^2+^; this mediates ecdysone exocytosis and thus results in the metamorphosis defect (Liu et al., 2019).

In CdsA-depleted testes, due to a hypomorphic mutation in *CdsA*, spermatogenesis is disrupted. The *CdsA* mutant shows a defect in spermatid individualization and enlargement of mitochondria and the axonemal sheath of the spermatids. A strong reduction in PI coupled with an increase in PA and TAG is observed. Although PI is decreased, this does not appear to result in a decrease in PI(4)P or PI(4,5)P_2_. Rather, the elevation of PA causes the male sterile phenotype as restoration of PA to normal levels rescues the spermatogenesis defect in *CdsA* mutants (Laurinyecz et al., 2016). Reduction in CdsA activity causes an elevation of total PA by sevenfold. How an increase in PA *per se* disrupts the complex process of spermatogenesis is not known. It is interesting to note that CdsA was previously described to function in rhabdomere biogenesis in the *Drosophila* eye, based on the analysis of the hypomorphic *Cds* allele (Raghu et al., 2009). In adult fly photoreceptors, *CdsA* mutant exhibits elevated levels of PA and depletion of PI(4,5)P_2_, which disrupts rhabdomere biogenesis and causes severe light-dependent photoreceptor degeneration (Wu et al., 1995; Raghu et al., 2009). Elevation of a single PA species (C34:2-PA) disrupted membrane transport to the apical domain in *Drosophila* photoreceptors and caused defective rhabdomere biogenesis in *CdsA* hypomorphic mutants (Raghu et al., 2009).

In summary, the studies in the different tissues of *Drosophila* reveal that disruption of *CdsA* (by either *CdsA RNAi* or by hypomorphic *CdsA* mutants) has a pleiotropic effect. Some of the phenotypes are attributed to increases in PA, others are due to defects in PLC and in PI3K signaling due to loss of PI levels, and the lipid storage phenotype is likely due to increases in TAG.

## Regulation of CDS1 and CDS2 Expression by Multiple Mechanisms

Recent studies indicate that both CDS1 and CDS2 expression levels are regulated and multiple mechanisms have been identified (Table 2). A recent study reported that treatment of several cell lines including HCT116, LoVo, and HepG cells with palmitic acid led to an increase in both CDS1 and CDS2 mRNA (Li et al., 2018). Palmitic acid also caused an increase in p53 and in SIRT6, a histone deacetylase. Knockdown of either p53 or SIRT6 abolished the increase in CDS1 and CDS2 mRNA leading to the conclusion that the increase in mRNA levels required both p53 and SIRT6. p53 and SIRT6 have been shown to interact, and the resulting complex was found to bind to the promoter region of *CDS1* and *CDS2*. SIRT6 was found to recruit RNA polymerase II and, importantly, this recruitment was dependent on p53 (Li et al., 2018).

Amongst its many functions, p53 regulates lipid metabolism including the acyl chain composition of PI (Naguib et al., 2015). Sirtuins are the guardians of mammalian lifespan and SIRT6 protects against pathological damage caused by diet-induced obesity (Kanfi et al., 2010; Kim et al., 2010; Giblin et al., 2014). In particular, SIRT6 is protective against accumulation of fats in the liver. In contrast, saturated fatty acids in the diet including palmitic acid induces intracellular lipid accumulation. In response to palmitic acid, the p53 and SIRT6 are induced and form a complex that binds to the promotors of CDS1 and CDS2, two enzymes required for PI synthesis (Li et al., 2018). Thus, wild type p53 not only shifts the PI acyl chain composition from mono-unsaturated to saturated, it also increases the enzymes that make CDP-DAG, the rate limiting step in PI synthesis. It should be noted that the study by Li et al. (2018) suggested that p53 and SIRT6 cooperate to regulate CL biosynthesis despite other known genes involved in CL synthesis were unaffected by palmitic acid treatment. Thus, their underlying assumption was that CDS1 and CDS2 were required for CL synthesis, not PI synthesis. It is more likely that CDS1 and CDS2 participate in PI synthesis. A deficiency in cellular PI is thought to promote non-alcoholic fatty liver disease (Michell, 2018) and an increase in PI levels by induction of CDS1 and CDS2 may be protective against accumulation of fats. In this context, it is interesting to note that gain of function mutations in p53 leads to profound changes in the fatty acid profile of PI toward less saturated acyl chains (Naguib et al., 2015). As described before, in many tissues and cell types, PI is highly enriched in one particular species: C18:0/C20:4.

Another regulator of CDS1 and CDS2 expression is the transcriptional coregulator, PGC-1α/β. PGC-1α/β is a member of a family of transcriptional coactivators that play key roles in the regulation of mitochondrial biogenesis and metabolism (Di et al., 2018). Expression of CDS1 mRNA was reduced in both PGC-1α^–/–^ and PGC-1β^–/–^ mice with the greatest reduction in the double knockout. Conversely, expression of PGC-1α or -1β independently increased CDS1 mRNA in neonatal rat cardiac myocytes nearly 10-fold (Lai et al., 2014). The *Cds1* promoter region was found to possess two conserved ERR binding site sequences and combined expression of PGC-1α or PGC-1β with ERR in differentiated C2C12 myoblasts resulted in synergistic transcriptional activation of the *Cds1* gene compared to PGC-1α or β on its own. Deletion of the ERR binding sites from the promoter region completely abolished the activating effects of PGC-1α. PGC-1α expression is highly inducible at the transcriptional level in response to a variety of upstream signaling pathways including energy deprivation, Ca^2+^, NO, and cAMP (Patten and Arany, 2012). PGC-1αβ^–/–^ hearts from mice show a mitochondrial cristae-stacking abnormality and a decrease in CL levels (Lai et al., 2014). Thus, PGC-1α induced CDS1 expression would suggest that ER-localized CDS1 might provide CDP-DAG for CL synthesis to mitochondria.

Cds1 mRNA expression is also regulated by ZEB1, a zinc finger E-Box transcriptional repressor (Gemmill et al., 2011). ZEB1 drives the epithelial to mesenchymal transition (EMT) through repression of epithelial genes (Gheldof et al., 2012). The expression of ZEB1 and Cds1 mRNA was monitored in 22 NSCLC (non-small cell lung cancer) cell-lines and an inverse relationship was observed (Gemmill et al., 2011). This result was validated in H358 cells; over-expression of ZEB1 decreased CDS1 mRNA whilst knockdown of ZEB1 increased CDS1 mRNA expression. The polarity of epithelial cells is maintained by high levels of PI(4,5)P_2_ at the apical surface and of PI(3,4,5)P_3_ at the basolateral surface (Comer and Parent, 2007; Martin-Belmonte and Mostov, 2007; Shewan et al., 2011). Thus, a decrease in CDS1 mRNA during mesenchymal transition would cause a reduction in PI levels aiding in this process.

Gene expression can be silenced by microRNAs (miRNAs) that function primarily by targeting the 3′-untranslated region (3′-UTR) of specific mRNAs. miR-16 can directly recognize the 3′-UTR of CDS2 and mediate the post-transcriptional inhibition of this gene (Yan et al., 2013). miR-16 is ubiquitously expressed and its loss has been linked to many types of cancers, such as chronic lymphocytic leukemia, prostate cancer and lung cancer (Pekarsky and Croce, 2019). Loss of miR-16 would increase CDS2 expression and it can be speculated that increased CDP-DAG would provide substrates to make PI(4,5)P_2_ and PI(3,4,5)P_3_ contributing toward the cancer phenotype.

AMP activated protein kinase (AMPK) controls energy homeostasis and deletion of AMPKα2, the main cardiac isoform, leads to decreases in CL levels by 25% and impairment of oxidative capacity due to defects at complex I of the respiratory chain (Athea et al., 2007). The mitochondria and myofibrils in myocytes are arranged in a regular ordered manner and the AMPKα2 knockout mice showed myofibrillar disorganization and irregular arrangement of the mitochondria. Surprisingly, the decrease in CL was accompanied by a decrease in CDS2 mRNA (Athea et al., 2007). However, CDS1 or TAMM41 were not examined leaving open the possibility that these enzymes may also be affected.

## Cdp-Dag Synthase in Bacteria

In bacteria, CDP-DAG serves as a precursor of the major phospholipids including PG, CL, PS, and PE (produced through decarboxylation of PS) (Icho et al., 1985; Sparrow and Raetz, 1985; Parsons and Rock, 2013). The *E. coli* CdsA, responsible for CDP-DAG formation, comprises nine transmembrane domains with a CDS signature motif (Sato et al., 2019; Sawasato et al., 2019; Figure 2A). *CdsA* is an essential gene, but mutants with decreased activity have been isolated that accumulate PA that leads to membrane dysfunction (Ganong et al., 1980; Ganong and Raetz, 1982).

In addition to the synthesis of the main lipids, CdsA is also required for the synthesis of a glycolipid, MPIase (membrane protein integrase). In this reaction, CDP-DAG, first synthesized by CdsA, incorporates GlcNAc-1-phosphate to produce GlcNAc-PP-DAG whilst associated with the enzyme. GlcNAc-PP-DAG is the precursor of MPIase. MPIase is composed of a glycan chain and DAG connected through a pyrophosphate linker and is required for integration of membrane proteins (Nishiyama et al., 2012; Sawasato et al., 2019). Thus, CdsA possesses two separate activities, the ability to make CDP-DAG and to make MPIase.

Recently, *YnbB* was identified as a *CdsA* paralog with a highly homologous C-terminal half (Figure 2A), where the catalytic site resides. Under *CdsA*-depleted conditions, YnbB over-production was able to restore MPIase, but not phospholipid levels. *YnbB* complemented the growth defect of the *CdsA* knockout when Tam41, the yeast mitochondrial enzyme, was co-expressed, indicating that YnbB possess activity for MPIase synthesis but not for phospholipid synthesis. The *YnbB* knockout exhibits no defects in phospholipid or MPIase biosynthesis indicating that CdsA normally fulfils both functions. However, when *CdsA* is deleted, PA accumulation and depletion of MPIase is observed. YnbB expression restores MPIase but not phospholipid biosynthesis. Likewise, Tam41 expression suppresses PA accumulation but is unable to restore MPIase synthesis.

Whilst the C-terminal half of YnbB is homologous to that of CdsA, no significant homology is found in the N-terminal halves (Figure 2A). Both CdsA and YnbB can make CDP-DAG *in vitro*. By creating chimeras of the two enzymes, it was revealed that the conserved C-terminal halves are similarly active in both phospholipid and MPIase synthesis, but that the N-terminal half of YnbB is not sufficient for phospholipid biosynthesis. In effect, the N-terminal half of CdsA is required to accelerate CDP-DAG synthesis (Sato et al., 2019).

YnbB is conserved among a wide range of bacteria strongly suggesting that it is a backup enzyme for MPIase biosynthesis, since MPIase is essential for membrane protein integration and therefore cell growth. Based on the crystal structure, *Tm*CdsA comprises three domains, namely an N-domain, M-domain and a C-domain (Figure 2A). The M-domain comprises the dimer interface, and the C-domain contains the active site. In the structure, the N-domain and the C-domain come close to form a gate into the lipid bilayer for CDP-DAG. Therefore, it was suggested the N-domain participates in the release of CDP-DAG. The N-domain of CdsA and YnbB are not homologous (Figure 2A) and this could contribute to the different rates of CDP-DAG biosynthesis. The eukaryotic homolog of CdsA, *Sc*CdsA or human CDS1 were able to complement *CdsA*-null bacteria provided that *Tam41* was also co-expressed. It would appear that *Sc*CdsA and human CDS1, but not Tam41, are also able to synthesize MPIase (Sawasato et al., 2019). The requirement of co-expression of Tam41 and the eukaryotic homolog of CdsA would suggest that the eukaryotic enzymes are less efficient in making CDP-DAG in a prokaryotic setting. An interesting point to emerge from these studies is whether MPIase is also made in eukaryotes by CDS enzymes and is required for integration of membrane proteins.

## Mutations in Bacterial Cdsa Cause Resistance to the Drug, Daptomycin

The anti-bacterial lipopeptide daptomycin (DAP) is used as a therapy against β-lactam-resistant *Streptococcus mitis/oralis* strains. The target of DAP is thought to be PG (Müller et al., 2016). Unlike other bacteria, *S. mitis/oralis* strains rapidly develop resistance to DAP due to loss of function mutations in *CdsA* (Mishra et al., 2017). DAP-resistant strains show loss of PG and CL and an increase in PA. Like other CdsA homologs, *CdsA* of *S. mitis/oralis* is predicted to possess nine transmembrane domains with a similar topology to *Tm*CdsA, with cytosolic binding loops (α and β), and the conserved CDS motif (Figure 2A). Mutation of Asp^222^ to Asn was found in the resistant mutant. Asp^222^ of *S. mitis/oralis* CdsA aligns with Asp^219^ of *Tm*CdsA, which forms part of a cation-binding Asp-Asp dyad essential for enzymatic activity.

## Cdp-Dag Synthase in Plants

In *Arabidopsis thaliana* CDS proteins are encoded by five genes, *CDS1*-*CDS5* (Haselier et al., 2010). In addition, *CDS2* and *CDS4* genes are alternatively spliced resulting in potentially 10 proteins. CDS1, CDS2 and CDS3 proteins are more similar to eukaryotic CDS than to prokaryote CDS, whilst CDS4 and CDS5 show the highest sequence similarity to the prokaryote, cyanobacteria. In addition, CDS4 and CDS5 contain cleavable N-terminal transit peptides for the import into plastids suggesting that CDS4 and CDS5 code for plastidial enzymes (plastids are derived from endosymbiotic cyanobacteria). CDS4 and CDS5 have redundant functions and only the double mutant shows a phenotype. Seedlings of the double mutant are not viable in soil, grow more slowly on agar-solidified medium with sucrose than wild-type plants, and develop yellow green leaves due to a reduction in chlorophyll. Although the size of the plastids in the double mutant are the same as wild type, there are severe defects in thylakoid structure. In the double mutant, PG biosynthesis is compromised and this is coupled to an increase in PA. PG is also required for the photosynthetic function contributing to the maintenance of chlorophyll-protein complexes in thylakoid membranes (Hagio et al., 2000; Sato et al., 2000). In the cyanobacterium, *Synechocystis* sp. PCC6803, mutants defective in the CDS gene show a decrease in PG and growth is repressed which can be rescued by exogenous PG (Sato et al., 2000). The Cds protein is 293 amino acids and exhibits a 38% identity with that of *E. coli* Cds, and like the *E. coli* Cds, lacks a hydrophilic N-terminus commonly present in the eukaryotic CDSs (Figure 2A).

CDS1 and CDS2 are constitutively expressed whilst CDS3 is only expressed in certain plant structures including stamens and mature pollens (Zhou et al., 2013). Both CDS1 and CDS2 are located at the ER and single mutants lacking either CDS1 or CDS2 are viable but the double mutant is not indicating redundancy. After germination, the double mutants failed to develop leaves. The double mutant seedlings showed a dramatic increase in PA levels accompanied by a decrease in PI levels but no significant change in PG levels. In summary, whilst CDS4 and CDS5 supply CDP-DAG for the synthesis of PG in plastids, CDS1, and CDS2 supply CDP-DAG for the synthesis of PI. In plants, PG is also made in microsomes and therefore its levels were also reduced in the CDS1/CDS2 knockouts, whilst CL was not affected (Zhou et al., 2013).

## Cds in Apicomplexa

CDS proteins of *Plasmodium falciparum* and *Toxoplasma gondii* both belong to the phylum Apicomplexa. These CDS proteins possess a long N-terminal extension compared with other eukaryotic CDS proteins (Shastri et al., 2010; Kong et al., 2017).

## Plasmodium falciparum

*Plasmodium falciparum* is a unicellular protozoan parasite and is the major cause of malaria in humans. *P. falciparum* require intense membrane synthesis when red cells are infected, and at this intraerythrocytic stage, glycerophospholipids originate from the *P. falciparum*-encoded machinery. Erythrocytes lack the machinery to make phospholipids. Synthesis of phospholipids in *P. falciparum* occurs through the same pathways as in higher eukaryotes. However, as in yeast, the malarial parasite has retained the prokaryotic system for PS synthesis from CDP-DAG. Synthesis of PI, the precursor for the polyphosphoinositides, is essential and the single CDS gene provides CDP-DAG for its synthesis.

The gene encoding CDS was isolated from *P. falciparum* based on sequence conservation to CDS from other organisms (Martin et al., 2000). Unlike other eukaryotic CDSs, *Pf*CDS is a large protein of 667 amino acids with a molecular weight of 78 kDa. Only the C-terminal 442 a.a. share 40% homology with eukaryotic CDSs. The long non-conserved N-terminal region of 245 a.a. is hydrophilic and cleaved during translation resulting in a 51 kDa protein that expresses CDS activity (Dechamps et al., 2010; Shastri et al., 2010) and a 28 kDa fragment. The conserved C-terminal fragment of *Pf*CDS is sufficient for enzyme function but insufficient to rescue *P. knowlesi* when its endogenous CDS is deleted. Rescue can only be effected by the full-length protein. *P. knowlesi* contains a CDS enzyme of 640 a.a. and is characterized by the N-terminal long extension similarly to *P. falciparum*. *Pk*CDS is an essential gene for *P. knowlesi* blood stage parasites and could be rescued by *Pf*CDS despite the fact that the N-terminal extension between *Pf*CDS and *Pk*CDS has only 26% identity. The N-terminal extension of CDS was found to be essential as rescue was not achievable with the truncated form, which contains the CDS activity (Shastri et al., 2010). Although the function of the cleaved 28 kDa fragment is not known, it is trafficked outside the parasite to the parasitophorous vacuole membrane.

Not all members of the Apicomplexa have long N-terminal extensions. It appears to be present in only those organisms that reside in their host cell with a parasitophorous vacuole, whereas CDS from other Apicocomplexa (e.g., *Babesia bovis*, *Theileria parva*, and *Crytosporidium parvum*) are similar to other eukaryotic CDS (Dechamps et al., 2010; Kong et al., 2017).

## Toxoplasma gondii

*Toxoplasma gondii* is a protozoan parasite and replicates within a vacuole in nucleated cells of virtually all warm-blooded organisms. For replication, phospholipid synthesis is essential and CDP-DAG is a major precursor for phospholipid synthesis. In *T. gondii*, two phylogenetically divergent CDS enzymes are present (Kong et al., 2017). The eukaryotic type *Tg*Cds1 that resides in the ER and prokaryotic type *Tg*Cds2 that resides in the apicoplast. Unlike the enzymes in mammals, yeast or *Drosophila* which are approximately ∼450 a.a. long (Figure 2A), *Tg*Cds1 and *Tg*Cds2 are just over 1000 a.a. due to an extensive N-terminal region (Kong et al., 2017). The apicoplast is a non-photosynthetic plastid-like organelle acquired by secondary endosymbiosis of red algae. *Tg*Cds2 orthologs have been identified in selected parasites such as *T. cruzi* and *L. major* but not in *P. falciparum* and *T. brucei* (Table 1). In *P. falciparum* and *T. brucei*, only one eukaryotic-type CDS has been identified. It should be noted that the mitochondrial CDS, TAMM41, is absent in protozoans (Kong et al., 2017).

*Tg*Cds1 and *Tg*Cds2 mutants are essential genes, and conditional knockout of *Tg*Cds1 impaired the synthesis of PI and PS, whilst knockdown of *Tg*Cds2 resulted in the selective impairment of PG. The mutants show severely reduced growth due to impaired replication and this could not be restored upon addition of exogenous lipids. The data suggests ER-derived CDP-DAG is utilized for PI synthesis in Golgi bodies, whilst CDP-DAG made in the apicoplast is utilized for making PG in mitochondria. Whilst apicoplasts can make PA, for PG synthesis CDP-DAG will have to be transferred to mitochondria where the enzymes are located. It is worth noting that membrane contact sites between the apicoplasts and the ER has been revealed by electron tomography in *T. gondii* (Tomova et al., 2009). Lipid transfer proteins that can bind and transfer CDP-DAG from one membrane compartment to another have yet to be identified.

## Eimeria falciformis

*Eimeria falciformis* is a mouse parasite with a complex lifestyle and undergoes the sporozoite stage that develops outside the host cells. Thus, the freely developing sporozite has to synthesize its own lipids. Phospholipid biogenesis is highly compartmentalized, spread primarily in the apicoplast, ER, mitochondrion and Golgi body. Like *T. gondii*, *E. falciformis* also contain two phylogenetically divergent CDS enzymes (Kong et al., 2017, 2018; Table 1). The ER contains the eukaryotic type *Ef*Cds1 whilst the prokaryotic type *Ef*Cds2 localizes to the apicoplast. PIS, the enzyme that makes PI from CDP-DAG, localizes to the Golgi body requiring the lipid transfer of CDP-DAG from the ER to the Golgi body. Likewise, the CDP-DAG made in the apicoplast by *Ef*CDS2 needs to be transferred to the mitochondria where the machinery to make PG and CL are located (Kong et al., 2018). Clearly, a significant amount of inter-organelle trafficking of CDP-DAG is required predicting the presence of multiple membrane contact sites. In other Apicocomplexa, membrane contact sites between the ER, Golgi complex, apicoplast and mitochondrion have been identified (Tomova et al., 2009).

## Cds in the Kinetoplastid, *Trypanosoma brucei*

*Trypanosoma brucei*, the causative agent of African sleeping sickness, expresses a VSG coat for protection from the host immune system. The VSG forms dimers that are inserted in the outer leaflet of the PM by glycosylphosphatidylinositol (GPI) anchors. GPI anchors are dependent on CDP-DAG for their synthesis. *T. brucei* has a single gene for *Cds* (Lilley et al., 2014). It is essential in the bloodstream form of the parasite. The *Tb*Cds conditional knockout shows morphological changes including a cell-cycle arrest due in part to kinetoplast segregation defects. Biochemical phenotyping of *Tb*CDS conditional knockout shows drastically altered lipid metabolism where reducing levels of PI detrimentally affected GPI biosynthesis. *Tb*CDS was shown to localize to the ER and Golgi, probably to provide CDP-DAG for PISs.

*Tb*CDS protein contains the typical CDS motif signature, and BLAST searches using typical prokaryotic CDS sequences recovered a putative second CDS gene in *T. cruzi*, *T. vivax*, and several *Leishmania* species, but not in *T. brucei*. All of these genes were more similar to the prokaryotic than the eukaryotic CDS genes. *T. Cruzi* appears to have lost its prokaryotic version of Cds that has been retained in other species (Table 1).

## Discovery of Tam41/Tamm41 as Possessing Cds Activity

Synthesis of CL in mitochondria is well-established (Horvath and Daum, 2013; Baile et al., 2014; Mejia et al., 2014; Schlame and Greenberg, 2017; Serricchio et al., 2018). Most of the key enzymes in the biosynthesis of CL, starting with PA, are characterized and are known to be localized in the matrix side of the inner mitochondrial membrane (Figure 1). For CL synthesis, the first reaction is the conversion of PA and CTP to CDP-DAG, and it has been assumed in some studies that the enzyme responsible for this conversion are the ER-localized CDS enzymes, CDS1 and CDS2 (Lai et al., 2014; Li et al., 2018). CDP-DAG is required for two steps in the synthesis of CL. In the first step, CDP-DAG is converted into PGP by the mitochondrial-localized enzyme, phosphatidylglycerol phosphate synthase (PGPS) (Kiyasu et al., 1963; Dzugasova et al., 1998; Kawasaki et al., 2001). A PGP phosphatase dephosphorylates PGP to form PG by unrelated enzymes, Gep4 (in yeast) or PTPMT1 (in mammals) localized in mitochondria (Osman et al., 2010; Zhang et al., 2011). Finally, a CL synthase (CLS), couples PG and another molecule of CDP-DAG to generate CL (Figure 1). CL undergoes remodeling of its fatty acid chains by a CL-specific phospholipase A and tafazzin (TAZ1), an acyltransferase to the final mature form of CL whose acyl chains are predominantly C18:2 (Schlame and Greenberg, 2017).

Uptake of newly synthesized mitochondrial proteins occur with the assistance of protein translocator complexes residing in the outer and inner mitochondrial membranes. In 2006, two studies in the yeast, *S. cerevisiae*, identified a mitochondrial protein that was required for the assembly and maintenance of the activity of the TIM23 complex. The protein was named Mmp37p (mitochondrial matrix protein of 37 kDa) (Gallas et al., 2006) or Tam41 (translocator assembly and maintenance 41) (Tamura et al., 2006). Tam41/Mmp37p was needed for the import of precursor proteins destined for the matrix and the inner membrane of the mitochondria via the TIM23 complex. Tam41 was characterized as a peripheral inner mitochondrial protein facing the matrix with homologs in many eukaryotic organisms. Tam41 was not part of the TIM23 complex, but was required for the maintenance and integrity of the TIM23 complex (Tamura et al., 2006). Furthermore, the assemblies of ADP/ATP carrier, complex III, and complex IV were also disrupted in *tam41*Δ mitochondria (Kutik et al., 2008).

Mitochondria lacking *Tam41* are temperature-sensitive for growth at elevated temperatures (Gallas et al., 2006; Tamura et al., 2006). Loss of *Tam41* revealed the lack of both PG and CL accompanied by an increase in PA in mitochondria (Kutik et al., 2008). Not surprisingly, Tam41 was subsequently found to possess CDP-DAG synthase activity (Tamura et al., 2013). Tam41 CDS activity was characterized using the purified protein; Tam41 from *S. cerevisiae* (*Sc*Tam41) shows maximal activity at pH 7–9, and requires divalent metal ions such as Mg^2+^, Mn^2+^, and Co^2+^. In mammals, CDS activity had been reported to be present in mitochondria (Mok et al., 1992, 1993), and recent studies have identified the mitochondrial activity to be due to TAMM41, the human homolog of Tam41 (Blunsom et al., 2017). A decrease in CL and a decline in oxygen consumption is observed in TAMM41-siRNA-treated H9c2 cardiomyoblasts. In Type I diabetic patients, DNA methylation of 780 mitochondrial proteins was examined and the *TAMM41* gene was found to be highly methylated in individuals with end-stage renal disease. Increased methylation of *TAMM41* gene would act to repress gene transcription suggesting that loss of TAMM41 function may be linked with the development of kidney disease (Swan et al., 2015).

Recently, the structure of Tam41 from *Schizosaccharomyces pombe* (*Sp*Tam41) was reported (Jiao et al., 2019). *Sp*Tam41 protein comprises 393 amino acid residues with a short mitochondrial pre-sequence at the N-terminal region (Figure 2B). Enzyme activity is optimal at pH 7.5 and divalent cations are essential for activity. The structure reveals that *Sp*Tam41 contains a nucleotidyltransferase (NTase) domain (residues 28–189) resembling the canonical fold of other NTases (Kuchta et al., 2009). Residues 190–319 folds into a winged helix domain, commonly found in nucleic acid binding proteins such as helicases, transcription factors and nucleic-acid-modifying enzymes (Harami et al., 2013). CTP binds to an “L”-shaped pocket sandwiched between the NTase and the winged helix domains. CTP and PA bind sequentially and both must be bound to the enzyme before either product (pyrophosphate and CDP-DAG) is released (Figure 3B). The protein that was crystallized lacked the 74 residues from the C-terminal region of *Sp*Tam41. However, bioinformatic analysis together with experiments revealed that *Sp*Tam41, like TAMM41, is tightly associated with membranes and can only be released upon treatment with Na_2_C0_3_ (pH 11) and this is due to a positively charged amphipathic helix at the C-terminal region (Figure 3B; Blunsom et al., 2017; Jiao et al., 2019).

## Functional Analysis of Tamm41 in Zebrafish

Biosynthesis of PG and CL takes place in the matrix side of the inner mitochondrial membrane and requires CDP-DAG as a central liponucleotide intermediate (Figure 1). The enzyme that makes PG and CL in mitochondria has been identified as Tam41/TAMM41 (Tamura et al., 2013; Blunsom et al., 2017). During zebrafish development, TAMM41 is enriched in the developing heart, and knockout of *Tamm41* driven by CRISPR, causes abnormal heart development, specifically a defect in heart valve formation. TAMM41 participates in regulating heart valve formation through mediating PINK1-dependent mitophagy (Yang et al., 2019). Examination of mitochondria of cardiac myocytes by electron microscopy showed that the mitochondria were much larger and more elongated. This was also observed in a heart cell line, AC16. The hyper-fused mitochondria is a predominant feature of these TAMM41-deficient cells. Interestingly, the level of CL was the same in the wild type and the TAMM41-depleted zebrafish. An increase in *Cds1* and *Pgs1* was observed, suggesting that mitochondrial TAMM41 deficiency can be compensated by CDS1. CDS1 resides in the ER and as such would require the transfer of CDP-DAG from the ER to the mitochondria (Figure 1).

To examine whether TAMM41 enzymatic activity was required for normal heart valve development, a mutant (Tamm41-D121A) was used in rescue experiments. In yeast, mutation of the residue D220A abolishes enzymatic activity; D121A is the corresponding mutation in zebrafish. Surprisingly, this mutant restored normal heart valve development indicating that CL biosynthesis was not required for this process. However, enzyme activity of the mutant protein was not analyzed.

In the *TAMM41*-deficient heart tissues that show increased numbers of enlarged mitochondria, mitochondrial proteins such as TOM20 and COXIV were increased. This was attributed to inhibition of PARK2-PINK1-dependent mitophagy. During mitophagy, PINK1 is stabilized at the mitochondrial membrane and phosphorylates the E3-ligase parkin (PARK2) which can then ubiquinate mitochondrial proteins. Silencing of *TAMM41* reduced the translocation of parkin thus reducing autophagy. TAMM41 interacted with PINK1 and stabilized PINK1 to allow mitophagy to take place. As the phenotype observed in zebrafish led to specific heart valve abnormalities, it was suggested that this was a tissue–specific role of *TAMM41* in heart development. It is noteworthy that TAMM41 is highly expressed in the developing heart.

## Do Cds Enzymes Provide Cdp-Dag to Mitochondria for Phosphatidylglycerol/Cardiolipin Synthesis?

One of the outstanding questions is the degree to which CDS1 and CDS2 contribute toward PG and CL synthesis. There is substantial evidence that both CDS1 and CDS2 can contribute to CL biosynthesis in heart (Lai et al., 2014). PGC-1 α and β are transcriptional regulators of mitochondrial metabolism and their loss in mouse heart causes a defect in CL biosynthesis resulting in mitochondrial ultrastructural abnormalities. The *CDS1* gene was downregulated whilst CDS2, PGS1, PTPMT1, and CLS1 mRNA were increased in PGC-1α/β^–/–^ mice. Importantly, the *CDS1* gene was shown to be a direct target for PGC-1α. The observation that CDS2 mRNA is increased together with the mRNA of the biosynthetic enzymes of the CL pathway suggests that CDS2 may contribute to CDP-DAG when CDS1 mRNA levels are reduced. No measurements of TAMM41 were done as the enzyme has not yet been identified.

Interestingly, CL content is also decreased in AMPKα2 knockout mice coupled to a decrease in CDS2 mRNA (Athea et al., 2007). The observation that knockout of different regulatory control mechanisms, i.e., PGC-1α/β or AMPKα2, can lead to a decrease in CL but is also linked to a decrease in CDS1 or CDS2 indicate that both CDS enzymes can contribute to CL synthesis. This would require mechanisms for transfer of CDP-DAG from the ER all the way to the inner membrane of the mitochondria crossing the outer membrane of the mitochondria and then transferred to the inner membrane (Figure 1). Such lipid transporters have not yet been identified.

Studies in yeast, where *Tam41* is deleted, a small amount of CL synthesis does occur suggesting that the yeast *CdsA* can contribute CDP-DAG for CL synthesis. Importantly, a greater increase in CL was observed when inositol was depleted simultaneously. Depletion of inositol would inhibit consumption of CDP-DAG by PIS making the CDP-DAG available for transfer from the ER to the mitochondria. Exogenous addition of a fluorescent CDP-DAG (NBD-CDP-DAG) to mitochondria also resulted in conversion into metabolites that were dependent on the presence of Pgs1. This indicated that exogenously added NBD-CDP-DAG could be transported to the inner mitochondrial membrane.

In several studies it has been noted that knockdown of CDS enzymes cause not only a decrease in PI but also PG, again arguing for a mechanism for the ER to supply mitochondria with CDP-DAG (Liu Y. et al., 2014; Laurinyecz et al., 2016; Qi et al., 2016; Kong et al., 2017). Close membrane contact sites between ER and mitochondria have been reported and this could provide a route for lipid transfer (Murley et al., 2015, 2017; Galmes et al., 2016; Horibata et al., 2017; Kawano et al., 2018). Other data also support the possibility that there is a pathway for CDP-DAG to traverse from the ER to the mitochondria. In *T. gondii*, there are two divergent CDS enzymes: the eukaryotic type, *Tg*Cds1, localized at the ER, and the prokaryotic type, *Tg*CDS2, localized at the apicoplast. Ablation of *TgCds2* resulted in the >80% reduction in PG biosynthesis, whilst ablation of *TgCds1* resulted in a decrease in PI and PS (Kong et al., 2017). As *T. gondii* lack Tam41, the prokaryotic-type CDS, *Tg*Cds2, in the apicoplast appears to provide the CDP-DAG to mitochondria for PG synthesis. The CDP-DAG made by *Tg*Cds1 in the ER appears to also translocate to the Golgi where *Tg*PIS is located to make PI. In *A. thaliana*, the two prokaryotic-type CDSs, CDS4, and CDS5 are also required for PG synthesis in the plastids; however, these enzymes are predicted to contain cleavable N-terminal transit peptides for the import into plastids.

These data all point to the presence of lipid transfer proteins that could mediate the transfer of CDP-DAG between different membrane compartments. Lipid transfer proteins that can bind and transfer CDP-DAG have yet to be identified. There are many lipid transfer proteins where the bound lipid remains to be characterized (Chiapparino et al., 2016; Wong et al., 2019). One potential candidate is the ER–mitochondria encounter structure (ERMES) complex found in yeast which are present at the ER- mitochondria contact sites (AhYoung et al., 2015). Three out of four ERMES complex proteins (Mmm1, Mdm12, and Mdm34) contain an SMP domain. The SMP domain can transport lipids by binding to the acyl chains of phospholipids rather than the headgroup. The ERMES complex is absent in mammals but a related protein, PDZD8, also contains an SMP domain and resides at ER-mitochondria contact sites (Hirabayashi et al., 2017).

## Concluding Remarks

Cytidine diphosphate diacylglycerol occupies a critical branch point in the synthesis of two lipids, PI and CL. Both lipids play essential roles in many aspects of cell function including cell signaling and mitochondrial function. The synthesis of these two lipids is compartmentalized in different membranes; ER for PI synthesis and mitochondria for PG and CL synthesis. Thus, the identification of two unrelated families of enzymes with CDS activity, CDS and TAMM41, which localize in specific compartments, would obviates the need for the transfer CDP-DAG from the ER to mitochondria under normal circumstances.

Studies where CDS1 and CDS2 levels have been decreased reveal that the cellular consequences can vary dramatically. The consequences of inhibition cause accumulation of PA and a decrease in PI levels. Both have consequences for cells that are manifested in a context-specific manner. Increased PA levels cause defects in membrane function whilst the decrease in PI levels impacts on both PLC and PI3K signaling. Moreover, in some cells, PA does not accumulate and is redirected toward other pathways leading to increases in TAG and thus accumulation of lipid droplets.

In mammalian cells, it appears that CDS2 may be the enzyme that in involved is PLC signaling based on the phenotype observed in zebrafish and its selectivity for 18:0/C20:4-PA. However, in cultured H9c2 cells, CDS1 appears to be more relevant as this is upregulated during vasopressin signaling when PI levels are dramatically decreased. It is possible that when PI levels are substantially reduced, CDS1 is more important as the synthesis of PI would not be limited by the availability of 18:0/C20:4-PA. It is also becoming clear that the expression levels of CDS enzymes are regulated in different conditions (Table 2). Since the formation of CDP-DAG is the rate limiting step in the synthesis of PI, changes in the levels of CDS enzymes are likely to regulate many of the signaling pathways including PLC and PI3K. The amount of PI(4,5)P_2_ in cells will determine both the extent and duration of signaling and therefore regulation of CDS expression under physiological or pathological conditions will have major consequences. Whether CDS enzymes are regulated post-translationally is not known, but the identification of residues that are phosphorylated or ubiquitylated implies that this is likely to be the case. Identification of the enzymes responsible for these modifications will provide a better understanding on regulation of these enzymes.

## Author Contributions

NB and SC wrote the review.

## Conflict of Interest

The authors declare that the research was conducted in the absence of any commercial or financial relationships that could be construed as a potential conflict of interest.

## Footnotes

1 https://www.thebiogrid.org

## Abbreviations

a.a.: amino acid
CarS: CDP-archeol synthase
CDP-DAG: CDP-diacylglycerol
CDS: CDP-diacylglycerol synthase
CL: cardiolipin
CLS: cardiolipin synthase
DAG: diacylglycerol
DAGK: diacylglycerol kinase
DGAT: diacylglycerol acyl transferase
DGGGP: 2,3-bis-O-geranylgeranyl *sn*-glycerol-phosphate
ERR: estrogen-related receptor
GPAT: glycerol phosphate acyl transferase
I(1,4,5)P_3_: inositol (1,4,5) triphosphate
Lipin: PA phosphatase
LPA: lyso-PA
LPAAT: lysoPA acyl transferase
LPIAT1: lyso-phosphatidylinositol acyltransferase 1
NBD: [(7-nitro-2-1,3-benzoxadiazol-4-yl) amino]
PA: phosphatidic acid
PC: phosphatidylcholine
PE: phosphatidylethanolamine
PG: phosphatidylglycerol
PGC-1 α / β: peroxisome proliferator-activated receptor γ-coactivator
PGP: phosphatidylglycerol phosphate
PGPS: PGP synthase
PI(3,4,5)P_3_: phosphatidylinositol (3,4,5)P_3_
PI(4)P: phosphatidylinositol (4) phosphate
PI(4,5)P_2_: phosphatidylinositol (4,5) bisphosphate
PI: phosphatidylinositol
PI3K: phosphoinositide 3-kinase
PIS: phosphatidylinositol synthase
PKC: protein kinase C
PLC: phospholipase C
PLD: phospholipase D
PM: plasma membrane
PPAR γ: peroxisome proliferator-activated receptor γ
PS: phosphatidylserine
PTPMT1: protein tyrosine phosphatase mitochondrial 1
SIRT6: sirtuin 6
TAG: triacylglycerol
Tam41, translocator assembly and maintenance protein: 41 kDa
TAMM41: mammalian Tam41
VEGFA: vascular endothelial growth factor A
VSG: variant surface glycoprotein.

## References

[B1] Acehan D. Malhotra A. Xu Y. Ren M. Stokes D. L. Schlame M. (2011). Cardiolipin affects the supramolecular organization of ATP synthase in mitochondria. *Biophys. J.* 100 2184–2192. 10.1016/j.bpj.2011.03.031 21539786 PMC3150712

[B2] AhYoung A. P. Jiang J. Zhang J. Khoi Dang X. Loo J. A. Zhou Z. H. (2015). Conserved SMP domains of the ERMES complex bind phospholipids and mediate tether assembly. *Proc. Natl. Acad. Sci. U.S.A.* 112 E3179–E3188. 10.1073/pnas.1422363112 26056272 PMC4485115

[B3] Alfonso Pecchio A. R. Cardozo Gizzi A. M. Renner M. L. Molina-Calavita M. Caputto B. L. (2011). c-Fos activates and physically interacts with specific enzymes of the pathway of synthesis of polyphosphoinositides. *Mol. Biol. Cell* 22 4716–4725. 10.1091/mbc.E11-03-0259 21998197 PMC3237616

[B4] Alhouayek M. Masquelier J. Muccioli G. G. (2018). Lysophosphatidylinositols, from cell membrane constituents to GPR55 Ligands. *Trends Pharmacol. Sci.* 39 586–604. 10.1016/j.tips.2018.02.011 29588059

[B5] Anderson K. E. Kielkowska A. Durrant T. N. Juvin V. Clark J. Stephens L. R. (2013). Lysophosphatidylinositol-acyltransferase-1 (LPIAT1) is required to maintain physiological levels of PtdIns and PtdInsP(2) in the mouse. *PLoS. One* 8:e58425. 10.1371/journal.pone.0058425 23472195 PMC3589398

[B6] Arimoto T. Takeishi Y. Takahashi H. Shishido T. Niizeki T. Koyama Y. (2006). Cardiac-specific overexpression of diacylglycerol kinase zeta prevents Gq protein-coupled receptor agonist-induced cardiac hypertrophy in transgenic mice. *Circulation* 113 60–66. 10.1161/circulationaha.105.560771 16380548

[B7] Athea Y. Viollet B. Mateo P. Rousseau D. Novotova M. Garnier A. (2007). AMP-activated protein kinase alpha2 deficiency affects cardiac cardiolipin homeostasis and mitochondrial function. *Diabetes* 56 786–794. 10.2337/db06-0187 17327449 PMC1955690

[B8] Baggelaar M. P. Maccarrone M. van der Stelt M. (2018). 2-Arachidonoylglycerol: a signaling lipid with manifold actions in the brain. *Prog. Lipid Res.* 71 1–17. 10.1016/j.plipres.2018.05.002 29751000

[B9] Baile M. G. Lu Y. W. Claypool S. M. (2014). The topology and regulation of cardiolipin biosynthesis and remodeling in yeast. *Chem. Phys. Lipids* 179 25–31. 10.1016/j.chemphyslip.2013.10.008 24184646 PMC3947685

[B10] Balla T. (2013). Phosphoinositides: tiny lipids with giant impact on cell regulation. *Physiol. Rev.* 93 1019–1137. 10.1152/physrev.00028.2012 23899561 PMC3962547

[B11] Barneda D. Cosulich S. Stephens L. Hawkins P. (2019). How is the acyl chain composition of phosphoinositides created and does it matter? *Biochem. Soc. Trans.* 47 1291–1305. 10.1042/BST20190205 31657437 PMC6824679

[B12] Barneda D. Planas-Iglesias J. Gaspar M. L. Mohammadyani D. Prasannan S. Dormann D. (2015). The brown adipocyte protein CIDEA promotes lipid droplet fusion via a phosphatidic acid-binding amphipathic helix. *Elife* 4:e07485. 10.7554/eLife.07485 26609809 PMC4755750

[B13] Blunsom N. J. Cockcroft S. (2020). Phosphatidylinositol synthesis at the endoplasmic reticulum. *Biochim. Biophys. Acta Mol. Cell Biol. Lipids* 1865 158471. 10.1016/j.bbalip.2019.05.015 31173893

[B14] Blunsom N. J. Gomez-Espinosa E. Ashlin T. G. Cockcroft S. (2017). Mitochondrial CDP-diacylglycerol synthase activity is due to the peripheral protein. TAMM41 and not due to the integral membrane protein, CDP-diacylglycerol synthase 1. *Biochim. Biophys. Acta* 1863 284–298. 10.1016/j.bbalip.2017.12.005 29253589 PMC5791848

[B15] Blunsom N. J. Gomez-Espinosa E. Ashlin T. G. Cockcroft S. (2019). Sustained phospholipase C stimulation of H9c2 cardiomyoblasts by vasopressin induces an increase in CDP-diacylglycerol synthase 1 (CDS1) through protein kinase C and cFos. *Biochim. Biophys. Acta Mol. Cell Biol. Lipids* 1864 1072–1082. 10.1016/j.bbalip.2019.03.002 30862571 PMC6495107

[B16] Bozelli J. C. Jr. Epand R. M. (2019a). Role of membrane shape in regulating the phosphatidylinositol cycle at contact sites. *Chem. Phys. Lipids* 221 24–29. 10.1016/j.chemphyslip.2019.03.002 30851248

[B17] Bozelli J. C. Jr. Epand R. M. (2019b). Specificity of Acyl chain composition of phosphatidylinositols. *Proteomics* 19:e1900138. 10.1002/pmic.201900138 31381272

[B18] Broekman M. J. Ward J. W. Marcus A. J. (1981). Fatty acid composition of phosphatidylinositol and phosphatidic acid in stimulated platelets. Persistence of arachidonyl-stearyl structure. *J. Biol. Chem.* 256 8271–8274. 6790536

[B19] Cardozo Gizzi A. M. Caputto B. L. (2013). Mechanistic insights into the nongenomic regulation of phospholipid synthesizing enzymes. *IUBMB Life* 65 584–592. 10.1002/iub.1173 23712998

[B20] Carman G. M. Kelley M. J. (1992). “CDPdiacylglycerol synthase from yeast,” in *Methods in Enzymology*, eds Dennis E. A. Vance e. E., (Cambridge, MA: Academic Press). 209 242–247. 10.1016/0076-6879(92)09030-7 1323038

[B21] Chiapparino A. Maeda K. Turei D. Saez-Rodriguez J. Gavin A. C. (2016). The orchestra of lipid-transfer proteins at the crossroads between metabolism and signaling. *Prog. Lipid Res.* 61 30–39. 10.1016/j.plipres.2015.10.004 26658141

[B22] Choy C. H. Han B. K. Botelho R. J. (2017). Phosphoinositide diversity, distribution, and effector function: stepping out of the box. *BioEssays* 39:1700121. 10.1002/bies.201700121 28977683

[B23] Chung J. Torta F. Masai K. Lucast L. Czapla H. Tanner L. B. (2015). Intracellular transport. PI4P/phosphatidylserine countertransport at ORP5- and ORP8-mediated ER-plasma membrane contacts. *Science* 349 428–432. 10.1126/science.aab1370 26206935 PMC4638224

[B24] Cockcroft S. Raghu P. (2016). Topological organisation of the phosphatidylinositol 4,5-bisphosphate-phospholipase C resynthesis cycle: PITPs bridge the ER-PM gap. *Biochem. J.* 473 4289–4310. 10.1042/bcj20160514c 27888240

[B25] Comer F. I. Parent C. A. (2007). Phosphoinositides specify polarity during epithelial organ development. *Cell* 128 239–240. 10.1016/j.cell.2007.01.010 17254962

[B26] D’Angelo G. Vicinanza M. Di Campli A. De Matteis M. A. (2008). The multiple roles of PtdIns(4)P – not just the precursor of PtdIns(4,5)P2. *J. Cell Sci* 121(Pt 12) 1955–1963. 10.1242/jcs.023630 18525025

[B27] Dechamps S. Shastri S. Wengelnik K. Vial H. J. (2010). Glycerophospholipid acquisition in *Plasmodium* – a puzzling assembly of biosynthetic pathways. *Int. J. Parasitol.* 40 1347–1365. 10.1016/j.ijpara.2010.05.008 20600072

[B28] Di W. Lv J. Jiang S. Lu C. Yang Z. Ma Z. (2018). PGC-1: the energetic regulator in cardiac metabolism. *Curr. Issues Mol. Biol.* 28 29–46. 10.21775/cimb.028.029 29388552

[B29] Di Paolo G. de Camilli P. (2006). Phosphoinositides in cell regulation and membrane dynamics. *Nature* 443 651–657. 10.1038/nature05185 17035995

[B30] D’Souza K. Epand R. M. (2015). The phosphatidylinositol synthase-catalyzed formation of phosphatidylinositol does not exhibit acyl chain specificity. *Biochemistry* 54 1151–1153. 10.1021/bi5015634 25633188

[B31] D’Souza K. Kim Y. J. Balla T. Epand R. M. (2014). Distinct properties of the two isoforms of CDP-diacylglycerol synthase. *Biochemistry* 53 7358–7367. 10.1021/bi501250m 25375833 PMC4255645

[B32] Dudek J. (2017). Role of cardiolipin in mitochondrial signaling pathways. *Front. Cell Dev. Biol.* 5:90. 10.3389/fcell.2017.00090 29034233 PMC5626828

[B33] Dudek J. Cheng I. F. Balleininger M. Vaz F. M. Streckfuss-Bomeke K. Hubscher D. (2013). Cardiolipin deficiency affects respiratory chain function and organization in an induced pluripotent stem cell model of Barth syndrome. *Stem Cell Res.* 11 806–819. 10.1016/j.scr.2013.05.005 23792436

[B34] Dzugasova V. Obernauerova M. Horvathova K. Vachova M. Zakova M. Subik J. (1998). Phosphatidylglycerolphosphate synthase encoded by the PEL1/PGS1 gene in *Saccharomyces cerevisiae* is localized in mitochondria and its expression is regulated by phospholipid precursors. *Curr. Genet.* 34 297–302. 10.1007/s002940050399 9799363

[B35] Epand R. M. So V. Jennings W. Khadka B. Gupta R. S. Lemaire M. (2016). Diacylglycerol Kinase-ε: properties and biological roles. *Front. Cell Dev. Biol.* 4:112. 10.3389/fcell.2016.00112 27803897 PMC5067486

[B36] Fei W. Shui G. Zhang Y. Krahmer N. Ferguson C. Kapterian T. S. (2011). A role for phosphatidic acid in the formation of “supersized” lipid droplets. *PLoS.Genet.* 7:e1002201. 10.1371/journal.pgen.1002201 21829381 PMC3145623

[B37] Gallas M. R. Dienhart M. K. Stuart R. A. Long R. M. (2006). Characterization of Mmp37p, a *Saccharomyces cerevisiae* mitochondrial matrix protein with a role in mitochondrial protein import. *Mol. Biol. Cell* 17 4051–4062. 10.1091/mbc.e06-04-0366 16790493 PMC1556384

[B38] Galmes R. Houcine A. van Vliet A. R. Agostinis P. Jackson C. L. Giordano F. (2016). ORP5/ORP8 localize to endoplasmic reticulum-mitochondria contacts and are involved in mitochondrial function. *EMBO Rep.* 17 800–810. 10.15252/embr.201541108 27113756 PMC5278607

[B39] Ganong B. R. Leonard J. M. Raetz C. R. (1980). Phosphatidic acid accumulation in the membranes of *Escherichia coli* mutants defective in CDP-diglyceride synthetase. *J. Biol. Chem.* 255 1623–1629. 6243645

[B40] Ganong B. R. Raetz C. R. (1982). Massive accumulation of phosphatidic acid in conditionally lethal CDP-diglyceride synthetase mutants and cytidine auxotrophs of *Escherichia coli*. *J. Biol. Chem.* 257 389–394. 6273438

[B41] Garner K. Hunt A. N. Koster G. Somerharju P. Grover E. Li M. (2012). Phosphatidylinositol transfer protein. Cytoplasmic 1 (PITPNC1) binds and transfers phosphatidic acid. *J. Biol. Chem.* 287 32263–32276. 10.1074/jbc.M112.375840 22822086 PMC3442557

[B42] Gemmill R. M. Roche J. Potiron V. A. Nasarre P. Mitas M. Coldren C. D. (2011). ZEB1-responsive genes in non-small cell lung cancer. *Cancer Lett.* 300 66–78. 10.1016/j.canlet.2010.09.007 20980099 PMC3337721

[B43] Gheldof A. Hulpiau P. van Roy F. De Craene B. Berx G. (2012). Evolutionary functional analysis and molecular regulation of the ZEB transcription factors. *Cell. Mol. Life Sci.* 69 2527–2541. 10.1007/s00018-012-0935-3 22349261 PMC11115101

[B44] Giblin W. Skinner M. E. Lombard D. B. (2014). Sirtuins: guardians of mammalian healthspan. *Trends Genet.* 30 271–286. 10.1016/j.tig.2014.04.007 24877878 PMC4077918

[B45] Gong J. Sun Z. Wu L. Xu W. Schieber N. Xu D. (2011). Fsp27 promotes lipid droplet growth by lipid exchange and transfer at lipid droplet contact sites. *J. Cell Biol.* 195 953–963. 10.1083/jcb.201104142 22144693 PMC3241734

[B46] Hagio M. Gombos Z. Varkonyi Z. Masamoto K. Sato N. Tsuzuki M. (2000). Direct evidence for requirement of phosphatidylglycerol in photosystem II of photosynthesis. *Plant Physiol.* 124 795–804. 10.1104/pp.124.2.795 11027727 PMC59183

[B47] Halford S. Dulai K. S. Daw S. C. Fitzgibbon J. Hunt D. M. (1998). Isolation and chromosomal localization of two human CDP-diacylglycerol synthase (CDS) genes. *Genomics* 54 140–144. 10.1006/geno.1998.5547 9806839

[B48] Halford S. Inglis S. Gwilliam R. Spencer P. Mohamed M. Ebenezer N. D. (2002). Genomic organization of human CDS2 and evaluation as a candidate gene for corneal hereditary endothelial dystrophy 2 on chromosome 20p13. *Exp. Eye Res.* 75 619–623. 10.1006/exer.2002.2052 12457874

[B49] Harami G. M. Gyimesi M. Kovacs M. (2013). From keys to bulldozers: expanding roles for winged helix domains in nucleic-acid-binding proteins. *Trends Biochem. Sci.* 38 364–371. 10.1016/j.tibs.2013.04.006 23768997

[B50] Hardie R. C. Liu C. H. Randall A. S. Sengupta S. (2015). *In vivo* tracking of phosphoinositides in *Drosophila* photoreceptors. *J. Cell Sci.* 128 4328–4340. 10.1242/jcs.180364 26483384 PMC4712823

[B51] Haselier A. Akbari H. Weth A. Baumgartner W. Frentzen M. (2010). Two closely related genes of *Arabidopsis* encode plastidial cytidinediphosphate diacylglycerol synthases essential for photoautotrophic growth. *Plant Physiol.* 153 1372–1384. 10.1104/pp.110.156422 20442275 PMC2899908

[B52] He Y. Yam C. Pomraning K. Chin J. S. Yew J. Y. Freitag M. (2014). Increase in cellular triacylglycerol content and emergence of large ER-associated lipid droplets in the absence of CDP-DG synthase function. *Mol. Biol. Cell* 25 4083–4095. 10.1091/mbc.E14-03-0832 25318672 PMC4263451

[B53] Heacock A. M. Uhler M. D. Agranoff B. W. (1996). Cloning of CDP-diacylglycerol synthase from a human neuronal cell line. *J. Neurochem.* 67 2200–2203. 10.1046/j.1471-4159.1996.67052200.x 8863531

[B54] Henry S. A. Kohlwein S. D. Carman G. M. (2012). Metabolism and regulation of glycerolipids in the yeast *Saccharomyces cerevisiae*. *Genetics* 190 317–349. 10.1534/genetics.111.130286 22345606 PMC3276621

[B55] Hill J. T. Demarest B. L. Bisgrove B. W. Gorsi B. Su Y.-C. Yost H. J. (2013). MMAPPR: mutation mapping analysis pipeline for pooled RNA-seq. *Genome Res.* 23 687–697. 10.1101/gr.146936.112 23299975 PMC3613585

[B56] Hille B. Dickson E. J. Kruse M. Vivas O. Suh B. C. (2015). Phosphoinositides regulate ion channels. *Biochim. Biophys. Acta* 1851 844–856. 10.1016/j.bbalip.2014.09.010 25241941 PMC4364932

[B57] Hirabayashi Y. Kwon S. K. Paek H. Pernice W. M. Paul M. A. Lee J. (2017). ER-mitochondria tethering by PDZD8 regulates Ca(2+) dynamics in mammalian neurons. *Science* 358 623–630. 10.1126/science.aan6009 29097544 PMC5818999

[B58] Hishikawa D. Hashidate T. Shimizu T. Shindou H. (2014). Diversity and function of membrane glycerophospholipids generated by the remodeling pathway in mammalian cells. *J. Lipid Res.* 55 799–807. 10.1194/jlr.R046094 24646950 PMC3995458

[B59] Horibata Y. Ando H. Satou M. Shimizu H. Mitsuhashi S. Shimizu Y. (2017). Identification of the N-terminal transmembrane domain of StarD7 and its importance for mitochondrial outer membrane localization and phosphatidylcholine transfer. *Sci. Rep.* 7:8793. 10.1038/s41598-017-09205-1 28821867 PMC5562819

[B60] Horvath S. E. Daum G. (2013). Lipids of mitochondria. *Prog. Lipid Res.* 52 590–614. 10.1016/j.plipres.2013.07.002 24007978

[B61] Hozumi Y. Fujiwara H. Kaneko K. Fujii S. Topham M. K. Watanabe M. (2017). Diacylglycerol kinase ε localizes to subsurface cisterns of cerebellar Purkinje cells. *Cell Tissue Res.* 368 441–458. 10.1007/s00441-017-2579-y 28191598

[B62] Icho T. Sparrow C. P. Raetz C. R. (1985). Molecular cloning and sequencing of the gene for CDP-diglyceride synthetase of *Escherichia coli*. *J. Biol. Chem.* 260 12078–12083. 2995358

[B63] Ikon N. Ryan R. O. (2017). Cardiolipin and mitochondrial cristae organization. *Biochim. Biophys. Acta Biomembr.* 1859 1156–1163. 10.1016/j.bbamem.2017.03.013 28336315 PMC5426559

[B64] Imae R. Inoue T. Nakasaki Y. Uchida Y. Ohba Y. Kono N. (2012). LYCAT, a homologue of C. *elegans* acl-8, acl-9, and acl-10, determines the fatty acid composition of phosphatidylinositol in mice. *J. Lipid Res.* 53 335–347. 10.1194/jlr.M018655 22172515 PMC3276457

[B65] Inglis-Broadgate S. L. Ocaka L. Banerjee R. Gaasenbeek M. Chapple J. P. Cheetham M. E. (2005). Isolation and characterization of murine Cds (CDP-diacylglycerol synthase) 1 and 2. *Gene* 356 19–31. 10.1016/j.gene.2005.04.037 16023307

[B66] Inloes J. M. Jing H. Cravatt B. F. (2018). The spastic paraplegia-associated phospholipase DDHD1 is a primary brain phosphatidylinositol lipase. *Biochemistry* 57 5759–5767. 10.1021/acs.biochem.8b00810 30221923 PMC6237197

[B67] Jain S. Caforio A. Fodran P. Lolkema J. S. Minnaard A. J. Driessen A. J. M. (2014). Identification of CDP-archaeol synthase, a missing link of ether lipid biosynthesis in Archaea. *Chem. Biol.* 21 1392–1401. 10.1016/j.chembiol.2014.07.022 25219966

[B68] Jiao H. Yin Y. Liu Z. (2019). Structures of the mitochondrial CDP-DAG synthase Tam41 suggest a potential lipid substrate pathway from membrane to the active site. *Structure 27*, 1258–1269.e4. 10.1016/j.str.2019.04.017 31178220

[B69] Kanfi Y. Peshti V. Gil R. Naiman S. Nahum L. Levin E. (2010). SIRT6 protects against pathological damage caused by diet-induced obesity. *Aging Cell* 9 162–173. 10.1111/j.1474-9726.2009.00544.x 20047575

[B70] Kawano S. Tamura Y. Kojima R. Bala S. Asai E. Michel A. H. (2018). Structure-function insights into direct lipid transfer between membranes by Mmm1-Mdm12 of ERMES. *J. Cell Biol.* 217 959–974. 10.1083/jcb.201704119 29279306 PMC5839780

[B71] Kawasaki K. Kuge O. Yamakawa Y. Nishijima M. (2001). Purification of phosphatidylglycerophosphate synthase from Chinese hamster ovary cells. *Biochem. J.* 354(Pt 1) 9–15. 10.1042/bj3540009 11171073 PMC1221622

[B72] Kelley M. J. Carman G. M. (1987). Purification and characterization of CDP-diacylglycerol synthase from *Saccharomyces cerevisiae*. *J. Biol. Chem.* 262 14563–14570. 2822695

[B73] Kim H. S. Xiao C. Wang R. H. Lahusen T. Xu X. Vassilopoulos A. (2010). Hepatic-specific disruption of SIRT6 in mice results in fatty liver formation due to enhanced glycolysis and triglyceride synthesis. *Cell Metab.* 12 224–236. 10.1016/j.cmet.2010.06.009 20816089 PMC2935915

[B74] Kim Y. J. Guzman-Hernandez M. L. Balla T. (2011). A highly dynamic ER-derived phosphatidylinositol-synthesizing organelle supplies phosphoinositides to cellular membranes. *Dev. Cell* 21 813–824. 10.1016/j.devcel.2011.09.005 22075145 PMC3235737

[B75] Kiyasu J. Y. Pieringer R. A. Paulus H. Kennedy E. P. (1963). The biosynthesis of phosphatidylglycerol. *J. Biol. Chem.* 238 2293–2298. 14033231

[B76] Kong P. Lehmann M. J. Helms J. B. Brouwers J. F. Gupta N. (2018). Lipid analysis of *Eimeria* sporozoites reveals exclusive phospholipids, a phylogenetic mosaic of endogenous synthesis, and a host-independent lifestyle. *Cell Discov.* 4:24. 10.1038/s41421-018-0023-4 29844921 PMC5964319

[B77] Kong P. Ufermann C. M. Zimmermann D. L. M. Yin Q. Suo X. Helms J. B. (2017). Two phylogenetically and compartmentally distinct CDP-diacylglycerol synthases cooperate for lipid biogenesis in *Toxoplasma gondii*. *J. Biol. Chem.* 292 7145–7159. 10.1074/jbc.M116.765487 28314772 PMC5409480

[B78] Kuchta K. Knizewski L. Wyrwicz L. S. Rychlewski L. Ginalski K. (2009). Comprehensive classification of nucleotidyltransferase fold proteins: identification of novel families and their representatives in human. *Nucleic Acids Res.* 37 7701–7714. 10.1093/nar/gkp854 19833706 PMC2794190

[B79] Kutik S. Rissler M. Guan X. L. Guiard B. Shui G. Gebert N. (2008). The translocator maintenance protein Tam41 is required for mitochondrial cardiolipin biosynthesis. *J. Cell Biol.* 183 1213–1221. 10.1083/jcb.200806048 19114592 PMC2606970

[B80] Lai L. Wang M. Martin O. J. Leone T. C. Vega R. B. Han X. (2014). A role for peroxisome proliferator-activated receptor gamma coactivator 1 (PGC-1) in the regulation of cardiac mitochondrial phospholipid biosynthesis. *J. Biol. Chem.* 289 2250–2259. 10.1074/jbc.M113.523654 24337569 PMC3900969

[B81] Laurinyecz B. Peter M. Vedelek V. Kovacs A. L. Juhasz G. Maroy P. (2016). Reduced expression of CDP-DAG synthase changes lipid composition and leads to male sterility in *Drosophila*. *Open Biol.* 6:50169. 10.1098/rsob.150169 26791243 PMC4736822

[B82] Lee H. C. Inoue T. Sasaki J. Kubo T. Matsuda S. Nakasaki Y. (2012). LPIAT1 regulates arachidonic acid content in phosphatidylinositol and is required for cortical lamination in mice. *Mol. Biol. Cell* 23 4689–4700. 10.1091/mbc.E12-09-0673 23097495 PMC3521678

[B83] Li M. Hou T. Gao T. Lu X. Yang Q. Zhu Q. (2018). p53 cooperates with SIRT6 to regulate cardiolipin de novo biosynthesis. *Cell Death Dis.* 9:941. 10.1038/s41419-018-0984-0 30237540 PMC6148051

[B84] Lilley A. C. Major L. Young S. Stark M. J. Smith T. K. (2014). The essential roles of cytidine diphosphate-diacylglycerol synthase in bloodstream form *Trypanosoma brucei*. *Mol. Microbiol.* 92 453–470. 10.1111/mmi.12553 24533860 PMC4114554

[B85] Liu C. H. Bollepalli M. K. Long S. V. Asteriti S. Tan J. Brill J. A. (2018). Genetic dissection of the phosphoinositide cycle in *Drosophila* photoreceptors. *J. Cell Sci.* 131:jcs214478. 10.1242/jcs.214478 29567856 PMC5963847

[B86] Liu X. Yin Y. Wu J. Liu Z. (2014). Structure and mechanism of an intramembrane liponucleotide synthetase central for phospholipid biosynthesis. *Nat. Commun.* 5:4244. 10.1038/ncomms5244 24968740 PMC4083444

[B87] Liu Y. Ji Y. Li X. Shui G. Huang X. (2019). Lipid storage regulator CdsA is essential for *Drosophila* metamorphosis. *J. Genet. Genomics* 46 231–234. 10.1016/j.jgg.2019.02.008 31072795

[B88] Liu Y. Wang W. Shui G. Huang X. (2014). CDP-diacylglycerol synthetase coordinates cell growth and fat storage through phosphatidylinositol metabolism and the insulin pathway. *PLoS Genet.* 10:e1004172. 10.1371/journal.pgen.1004172 24603715 PMC3945474

[B89] Lombard J. López-García P. Moreira D. (2012). The early evolution of lipid membranes and the three domains of life. *Nat. Rev. Microbiol.* 10 507–515. 10.1038/nrmicro2815 22683881

[B90] López-Lara I. M. Geiger O. (2017). Bacterial lipid diversity. *Biochim. Biophys. Acta Mol. Cell Biol. Lipids* 1862 1287–1299. 27760387 10.1016/j.bbalip.2016.10.007

[B91] Lung M. Shulga Y. V. Ivanova P. T. Myers D. S. Milne S. B. Brown H. A. (2009). Diacylglycerol kinase epsilon is selective for both acyl chains of phosphatidic acid or diacylglycerol. *J. Biol. Chem.* 284 31062–31073. 10.1074/jbc.M109.050617 19744926 PMC2781506

[B92] Lykidis A. (2007). Comparative genomics and evolution of eukaryotic phospholipid biosynthesis. *Prog. Lipid Res.* 46 171–199. 10.1016/j.plipres.2007.03.003 17512056

[B93] Lykidis A. Jackson P. D. Rock C. O. Jackowski S. (1997). The role of CDP-diacylglycerol synthase and phosphatidylinositol synthase activity levels in the regulation of cellular phosphatidylinositol content. *J. Biol. Chem.* 272 33402–33409. 10.1074/jbc.272.52.33402 9407135

[B94] MacDonald G. Baker R. R. Thompson W. (1975). Selective synthesis of molecular classes of phosphatidic acid, diacylglycerol and phosphatidylinositol in rat brain. *J. Neurochem.* 24 655–661. 10.1111/j.1471-4159.1975.tb11658.x 1123618

[B95] Maguire J. J. Tyurina Y. Y. Mohammadyani D. Kapralov A. A. Anthonymuthu T. S. Qu F. (2017). Known unknowns of cardiolipin signaling: the best is yet to come. *Biochim. Biophys. Acta Mol. Cell Biol. Lipids* 1862 8–24. 10.1016/j.bbalip.2016.08.001 27498292 PMC5323096

[B96] Maillet M. van Berlo J. H. Molkentin J. D. (2013). Molecular basis of physiological heart growth: fundamental concepts and new players. *Nat. Rev. Mol. Cell Biol.* 14 38–48. 10.1038/nrm3495 23258295 PMC4416212

[B97] Martin D. Gannoun-Zaki L. Bonnefoy S. Eldin P. Wengelnik K. Vial H. (2000). Characterization of *Plasmodium falciparum* CDP-diacylglycerol synthase, a proteolytically cleaved enzyme. *Mol. Biochem. Parasitol.* 110 93–105. 10.1016/s0166-6851(00)00260-7 10989148

[B98] Martin-Belmonte F. Mostov K. (2007). Phosphoinsositides cotrol epithelial development. *Cell Cycle* 6 1957–1961. 17712229 10.4161/cc.6.16.4583

[B99] Mejia E. M. Nguyen H. Hatch G. M. (2014). Mammalian cardiolipin biosynthesis. *Chem. Phys. Lipids* 179 11–16. 10.1016/j.chemphyslip.2013.10.001 24144810

[B100] Mercade A. Sanchez A. Folch J. M. (2007). Characterization and physical mapping of the porcine CDS1 and CDS2 genes. *Anim. Biotechnol.* 18 23–35. 17364441 10.1080/10495390601091073

[B101] Mérida I. Andrada E. Gharbi S. I. Ávila-Flores A. (2015). Redundant and specialized roles for diacylglycerol kinases α and ζ in the control of T cell functions. *Sci. Signal.* 8 re6. 10.1126/scisignal.aaa0974 25921290

[B102] Michell R. H. (1975). Inositol phospholipids in cell surface receptor function. *Biochim. Biophys. Acta* 415 81–147. 10.1016/0304-4157(75)90017-9 164246

[B103] Michell R. H. (2018). Do inositol supplements enhance phosphatidylinositol supply and thus support endoplasmic reticulum function? *Br. J. Nutr.* 120 301–316. 10.1017/S0007114518000946 29859544

[B104] Mishra N. N. Tran T. T. Seepersaud R. Garcia-de-la-Maria C. Faull K. Yoon A. (2017). Perturbations of phosphatidate cytidylyltransferase (CdsA) mediate daptomycin resistance in *Streptococcus* mitis/oralis by a novel mechanism. *Antimicrob. Agents Chemother.* 61:e02435-16. 10.1128/AAC.02435-16 28115347 PMC5365703

[B105] Mok A. Y. McDougall G. E. McMurray W. C. (1992). CDP-diacylglycerol synthesis in rat liver mitochondria. *FEBS Lett.* 312 236–240. 10.1016/0014-5793(92)80942-a 1330695

[B106] Mok A. Y. McDougall G. E. McMurray W. C. (1993). Comparative studies of CDP-diacylglycerol synthase in rat liver mitochondria and microsomes. *Biochem. Cell Biol.* 71 183–189. 10.1139/o93-029 8398077

[B107] Moser von Filseck J. Vanni S. Mesmin B. Antonny B. Drin G. (2015). A phosphatidylinositol-4-phosphate powered exchange mechanism to create a lipid gradient between membranes. *Nat. Commun.* 6:6671. 10.1038/ncomms7671 25849868

[B108] Motrich R. D. Castro G. M. Caputto B. L. (2013). Old players with a newly defined function: Fra-1 and c-Fos support growth of human malignant breast tumors by activating membrane biogenesis at the cytoplasm. *PLoS One* 8:e53211. 10.1371/journal.pone.0053211 23301044 PMC3534677

[B109] Mujalli A. Chicanne G. Bertrand-Michel J. Viars F. Stephens L. Hawkins P. (2018). Profiling of phosphoinositide molecular species in human and mouse platelets identifies new species increasing following stimulation. *Biochim. Biophys. Acta* 1863 1121–1131. 10.1016/j.bbalip.2018.06.009 29902570

[B110] Müller A. Wenzel M. Strahl H. Grein F. Saaki T. N. V. Kohl B. (2016). Daptomycin inhibits cell envelope synthesis by interfering with fluid membrane microdomains. *Proc. Natl. Acad. Sci. U.S.A.* 113 E7077–E7086. 10.1073/pnas.1611173113 27791134 PMC5111643

[B111] Murley A. Sarsam R. D. Toulmay A. Yamada J. Prinz W. A. Nunnari J. (2015). Ltc1 is an ER-localized sterol transporter and a component of ER-mitochondria and ER-vacuole contacts. *J. Cell Biol.* 209 539–548. 10.1083/jcb.201502033 25987606 PMC4442815

[B112] Murley A. Yamada J. Niles B. J. Toulmay A. Prinz W. A. Powers T. (2017). Sterol transporters at membrane contact sites regulate TORC1 and TORC2 signaling. *J. Cell Biol.* 216 2679–2689. 10.1083/jcb.201610032 28774891 PMC5584152

[B113] Naguib A. Bencze G. Engle D. D. Chio I. I. Herzka T. Watrud K. (2015). p53 mutations change phosphatidylinositol acyl chain composition. *Cell Rep.* 10 8–19. 10.1016/j.celrep.2014.12.010 25543136 PMC4287966

[B114] Nakagawa Y. Rustow B. Rabe H. Kunze D. Waku K. (1989). The de novo synthesis of molecular species of phosphatidylinositol from endogenously labeled CDP diacylglycerol in alveolar macrophage microsomes. *Arch. Biochem. Biophys.* 268 559–566. 10.1016/0003-9861(89)90323-8 2913948

[B115] Nakano T. Matsui H. Tanaka T. Hozumi Y. Iseki K. Kawamae K. (2016). Arachidonoyl-specific diacylglycerol kinase ε and the endoplasmic reticulum. *Front. Cell Dev. Biol.* 4:132 10.3389/fcell.2016.00132 PMC5114243 27917381

[B116] Niizeki T. Takeishi Y. Kitahara T. Arimoto T. Koyama Y. Goto K. (2008). Diacylglycerol kinase zeta rescues G alpha q-induced heart failure in transgenic mice. *Circ. J.* 72 309–317. 10.1253/circj.72.309 18219172

[B117] Nishiyama K. Maeda M. Yanagisawa K. Nagase R. Komura H. Iwashita T. (2012). MPIase is a glycolipozyme essential for membrane protein integration. *Nat. Commun.* 3:1260. 10.1038/ncomms2267 23232390 PMC3535364

[B118] Osman C. Haag M. Wieland F. T. Brugger B. Langer T. (2010). A mitochondrial phosphatase required for cardiolipin biosynthesis: the PGP phosphatase Gep4. *EMBO J.* 29 1976–1987. 10.1038/emboj.2010.98 20485265 PMC2892375

[B119] Pan W. Pham V. N. Stratman A. N. Castranova D. Kamei M. Kidd K. R. (2012). CDP-diacylglycerol synthetase-controlled phosphoinositide availability limits VEGFA signaling and vascular morphogenesis. *Blood* 120 489–498. 10.1182/blood-2012-02-408328 22649102 PMC3398756

[B120] Parsons J. B. Rock C. O. (2013). Bacterial lipids: metabolism and membrane homeostasis. *Prog. Lipid Res.* 52 249–276. 10.1016/j.plipres.2013.02.002 23500459 PMC3665635

[B121] Patten I. S. Arany Z. (2012). PGC-1 coactivators in the cardiovascular system. *Trends Endocrinol. Metab.* 23 90–97. 10.1016/j.tem.2011.09.007 22047951

[B122] Pekarsky Y. Croce C. M. (2019). Noncoding RNA genes in cancer pathogenesis. *Adv. Biol. Regul.* 71 219–223. 10.1016/j.jbior.2018.12.002 30611710 PMC6800108

[B123] Pettitt T. R. Martin A. Horton T. Liossis C. Lord J. M. Wakelam M. J. (1997). Diacylglycerol and phosphatidate generated by phospholipases C and D, respectively, have distinct fatty acid compositions and functions. Phospholipase D-derived diacylglycerol does not activate protein kinase C in porcine aortic endothelial cells. *J. Biol. Chem.* 272 17354–17359. 10.1074/jbc.272.28.17354 9211874

[B124] Prescott S. M. Majerus P. W. (1981). The fatty acid composition of phosphatidylinositol from thrombin- stimulated human platelets. *J. Biol. Chem.* 256 579–582. 7451460

[B125] Qi Y. Kapterian T. S. Du X. Ma Q. Fei W. Zhang Y. (2016). CDP-diacylglycerol synthases regulate the growth of lipid droplets and adipocyte development. *J. Lipid Res.* 57 767–780. 10.1194/jlr.M060574 26946540 PMC4847625

[B126] Racca A. C. Prucca C. G. Caputto B. L. (2019). Fra-1 and c-Fos N-terminal deletion mutants impair breast tumor cell proliferation by blocking lipid synthesis activation. *Front. Oncol.* 9:544. 10.3389/fonc.2019.00544 31275861 PMC6593343

[B127] Raghu P. Coessens E. Manifava M. Georgiev P. Pettitt T. Wood E. (2009). Rhabdomere biogenesis in *Drosophila* photoreceptors is acutely sensitive to phosphatidic acid levels. *J. Cell Biol.* 185 129–145. 10.1083/jcb.200807027 19349583 PMC2700502

[B128] Raghu P. Joseph A. Krishnan H. Singh P. Saha S. (2019). Phosphoinositides: regulators of nervous system function in health and disease. *Front. Mol. Neurosci.* 12:208. 10.3389/fnmol.2019.00208 31507376 PMC6716428

[B129] Ren S. Caforio A. Yang Q. Sun B. Yu F. Zhu X. (2017). Structural and mechanistic insights into the biosynthesis of CDP-archaeol in membranes. *Cell Res.* 27 1378–1391. 10.1038/cr.2017.122 28961231 PMC5674157

[B130] Robinson C. V. Rohacs T. Hansen S. B. (2019). Tools for understanding nanoscale lipid regulation of ion channels. *Trends Biochem. Sci.* 44 795–806. 10.1016/j.tibs.2019.04.001 31060927 PMC6729126

[B131] Rohit S. Aniruddha P. Padinjat R. Sandeep K. (2018). Evidence of sinks and sources in the phospholipase C−activated PIP2 cycle. *FEBS Lett.* 592 962–972. 10.1002/1873-3468.12998 29427502

[B132] Romanauska A. Kohler A. (2018). The inner nuclear membrane is a metabolically active territory that generates nuclear lipid droplets. *Cell* 174 700–715.e18. 10.1016/j.cell.2018.05.047 29937227 PMC6371920

[B133] Saito S. Goto K. Tonosaki A. Kondo H. (1997). Gene cloning and characterization of CDP-diacylglycerol synthase from rat brain. *J. Biol. Chem.* 272 9503–9509. 10.1074/jbc.272.14.9503 9083091

[B134] Sakane F. Mizuno S. Takahashi D. Sakai H. (2018). Where do substrates of diacylglycerol kinases come from? Diacylglycerol kinases utilize diacylglycerol species supplied from phosphatidylinositol turnover-independent pathways. *Adv. Biol. Regul.* 67 101–108. 10.1016/j.jbior.2017.09.003 28918129

[B135] Sanjuan M. A. Jones D. R. Izquierdo M. Merida I. (2001). Role of diacylglycerol kinase alpha in the attenuation of receptor signaling. *J. Cell Biol.* 153 207–220. 10.1083/jcb.153.1.207 11285286 PMC2185527

[B136] Sato N. Hagio M. Wada H. Tsuzuki M. (2000). Requirement of phosphatidylglycerol for photosynthetic function in thylakoid membranes. *Proc. Natl. Acad. Sci. U.S.A.* 97 10655–10660. 10.1073/pnas.97.19.10655 10984546 PMC27080

[B137] Sato R. Sawasato K. Nishiyama K. I. (2019). YnbB is a CdsA paralogue dedicated to biosynthesis of glycolipid MPIase involved in membrane protein integration. *Biochem. Biophys. Res. Commun.* 510 636–642. 10.1016/j.bbrc.2019.01.145 30739787

[B138] Sawasato K. Sato R. Nishikawa H. Iimura N. Kamemoto Y. Fujikawa K. (2019). CdsA is involved in biosynthesis of glycolipid MPIase essential for membrane protein integration *in vivo*. *Sci. Rep.* 9:1372. 10.1038/s41598-018-37809-8 30718729 PMC6362211

[B139] Schlame M. Greenberg M. L. (2017). Biosynthesis, remodeling and turnover of mitochondrial cardiolipin. *Biochim. Biophys. Acta Mol. Cell Biol. Lipids* 1862 3–7. 10.1016/j.bbalip.2016.08.010 27556952 PMC5125896

[B140] Serricchio M. Vissa A. Kim P. K. Yip C. M. McQuibban G. A. (2018). Cardiolipin synthesizing enzymes form a complex that interacts with cardiolipin-dependent membrane organizing proteins. *Biochim. Biophys. Acta Mol. Cell Biol. Lipids* 1863 447–457. 10.1016/j.bbalip.2018.01.007 29343430

[B141] Shastri S. Zeeman A. M. Berry L. Verburgh R. J. Braun-Breton C. Thomas A. W. (2010). *Plasmodium* CDP-DAG synthase: an atypical gene with an essential N-terminal extension. *Int. J. Parasitol.* 40 1257–1268. 10.1016/j.ijpara.2010.03.006 20385136

[B142] Shen H. Dowhan W. (1997). Regulation of phospholipid biosynthetic enzymes by the level of CDP-diacylglycerol synthase activity. *J. Biol. Chem.* 272 11215–11220. 10.1074/jbc.272.17.11215 9111022

[B143] Shen H. Heacock P. N. Clancey C. J. Dowhan W. (1996). The CDS1 gene encoding CDP-diacylglycerol synthase in *Saccharomyces cerevisiae* is essential for cell growth. *J. Biol. Chem.* 271 789–795. 10.1074/jbc.271.2.789 8557688

[B144] Shewan A. Eastburn D. J. Mostov K. (2011). Phosphoinositides in cell architecture. *Cold Spring Harb. Perspect. Biol.* 3:a004796. 10.1101/cshperspect.a004796 21576256 PMC3140688

[B145] Shulga Y. V. Topham M. K. Epand R. M. (2011). Regulation and functions of diacylglycerol kinases. *Chem. Rev.* 111 6186–6208. 10.1021/cr1004106 21800853

[B146] Singal T. Dhalla N. S. Tappia P. S. (2009). Regulation of c-Fos and c-Jun gene expression by phospholipase C activity in adult cardiomyocytes. *Mol. Cell. Biochem.* 327 229–239. 10.1007/s11010-009-0061-1 19225867

[B147] Sparrow C. P. Raetz C. R. (1985). Purification and properties of the membrane-bound CDP-diglyceride synthetase from *Escherichia coli*. *J. Biol. Chem.* 260 12084–12091. 2995359

[B148] Swan E. J. Maxwell A. P. McKnight A. J. (2015). Distinct methylation patterns in genes that affect mitochondrial function are associated with kidney disease in blood-derived DNA from individuals with Type 1 diabetes. *Diabet. Med.* 32 1110–1115. 10.1111/dme.12775 25850930

[B149] Takeishi Y. Goto K. Kubota I. (2007). Role of diacylglycerol kinase in cellular regulatory processes: a new regulator for cardiomyocyte hypertrophy. *Pharmacol. Ther.* 115 352–359. 10.1016/j.pharmthera.2007.04.010 17659347

[B150] Tamura Y. Harada Y. Nishikawa S. I. Yamano K. Kamiya M. Shiota T. (2013). Tam41 is a CDP-diacylglycerol synthase required for cardiolipin biosynthesis in mitochondria. *Cell Metab.* 17 1–10. 23623749 10.1016/j.cmet.2013.03.018 PMC3654088

[B151] Tamura Y. Harada Y. Yamano K. Watanabe K. Ishikawa D. Ohshima C. (2006). Identification of Tam41 maintaining integrity of the TIM23 protein translocator complex in mitochondria. *J. Cell Biol.* 174 631–637. 10.1083/jcb.200603087 16943180 PMC2064306

[B152] Tomova C. Humbel B. M. Geerts W. J. Entzeroth R. Holthuis J. C. Verkleij A. J. (2009). Membrane contact sites between apicoplast and ER in *Toxoplasma gondii* revealed by electron tomography. *Traffic* 10 1471–1480. 10.1111/j.1600-0854.2009.00954.x 19602198

[B153] Traynor-Kaplan A. Kruse M. Dickson E. J. Dai G. Vivas O. Yu H. (2017). Fatty-acyl chain profiles of cellular phosphoinositides. *Biochim. Biophys. Acta Mol. Cell Biol. Lipids* 1862 513–522. 10.1016/j.bbalip.2017.02.002 28189644 PMC5392126

[B154] Vanhaesebroeck B. Stephens L. Hawkins P. (2012). PI3K signalling: the path to discovery and understanding. *Nat. Rev. Mol. Cell Biol.* 13 195–203. 10.1038/nrm3290 22358332

[B155] Volta M. Bulfone A. Gattuso C. Rossi E. Mariani M. Consalez G. G. (1999). Identification and characterization of CDS2, a mammalian homolog of the *Drosophila* CDP-diacylglycerol synthase gene. *Genomics* 55 68–77. 10.1006/geno.1998.5610 9889000

[B156] Weeks R. Dowhan W. Shen H. Balantac N. Meengs B. Nudelman E. (1997). Isolation and expression of an isoform of human CDP-diacylglycerol synthase cDNA. *DNA Cell Biol.* 16 281–289. 10.1089/dna.1997.16.281 9115637

[B157] Wong L. H. Gatta A. T. Levine T. P. (2019). Lipid transfer proteins: the lipid commute via shuttles, bridges and tubes. *Nat. Rev. Mol. Cell Biol.* 20 85–101. 10.1038/s41580-018-0071-5 30337668

[B158] Wu L. Niemeyer B. Colley N. Socolich M. Zuker C. S. (1995). Regulation of PLC-mediated signalling *in vivo* by CDP-diacylglycerol synthase. *Nature* 373 216–222. 10.1038/373216a0 7816135

[B159] Xu Y. Mak H. Y. Lukmantara I. Li Y. E. Hoehn K. L. Huang X. (2019). CDP-DAG Synthase 1 and 2 regulate lipid droplet growth through distinct mechanisms. *J. Biol. Chem.* 294 16740–16755. 10.1074/jbc.RA119.009992 31548309 PMC6851299

[B160] Yan X. Liang H. Deng T. Zhu K. Zhang S. Wang N. (2013). The identification of novel targets of miR-16 and characterization of their biological functions in cancer cells. *Mol. Cancer* 12:92. 10.1186/1476-4598-12-92 23941513 PMC3751425

[B161] Yang R. M. Tao J. Zhan M. Yuan H. Wang H. H. Chen S. J. (2019). TAMM41 is required for heart valve differentiation via regulation of PINK-PARK2 dependent mitophagy. *Cell Death Differ.* 26 2430–2446. 10.1038/s41418-019-0311-z 30824836 PMC6888875

[B162] Yang Y. Lee M. Fairn G. D. (2018). Phospholipid subcellular localization and dynamics. *J. Biol. Chem.* 293 6230–6240. 10.1074/jbc.r117.000582 29588369 PMC5925819

[B163] Yen H. Y. Hoi K. K. Liko I. Hedger G. Horrell M. R. Song W. (2018). PtdIns(4,5)P2 stabilizes active states of GPCRs and enhances selectivity of G-protein coupling. *Nature* 559 423–427. 10.1038/s41586-018-0325-6 29995853 PMC6059376

[B164] Zhang J. Guan Z. Murphy A. N. Wiley S. E. Perkins G. A. Worby C. A. (2011). Mitochondrial phosphatase PTPMT1 is essential for cardiolipin biosynthesis. *Cell Metab.* 13 690–700. 10.1016/j.cmet.2011.04.007 21641550 PMC3119201

[B165] Zhao W. Cao L. Ying H. Zhang W. Li D. Zhu X. (2019). Endothelial CDS2 deficiency causes VEGFA-mediated vascular regression and tumor inhibition. *Cell Res.* 29 895–910. 10.1038/s41422-019-0229-5 31501519 PMC6889172

[B166] Zhou Y. Peisker H. Weth A. Baumgartner W. Dormann P. Frentzen M. (2013). Extraplastidial cytidinediphosphate diacylglycerol synthase activity is required for vegetative development in *Arabidopsis thaliana*. *Plant J.* 75 867–879. 10.1111/tpj.12248 23711240

